# Deep-Learning-Driven High Spatial Resolution Attenuation Imaging for Ultrasound Tomography (AI-UT)

**DOI:** 10.1109/TUFFC.2025.3592578

**Published:** 2025-09

**Authors:** Mingrui Liu, Zhengchang Kou, James W. Wiskin, Gregory J. Czarnota, Michael L. Oelze

**Affiliations:** Department of Electrical and Computer Engineering, Beckman Institute, Grainger College of Engineering, University of Illinois at Urbana–Champaign, Urbana, IL 61801 USA.; Beckman Institute, University of Illinois at Urbana–Champaign, Urbana, IL 61801 USA.; QT Imaging Inc., Novato, CA 94949 USA.; Sunnybrook Health Sciences Centre, Toronto, ON M4N 3M5, Canada.; Department of Electrical and Computer Engineering, Beckman Institute, Grainger College of Engineering, and the Carle Illinois College of Medicine, University of Illinois at Urbana–Champaign, Urbana, IL 61801 USA.

**Keywords:** Attenuation imaging, breast imaging, deep learning (DL), quantitative ultrasound (QUS), ultrasound tomography

## Abstract

Ultrasonic attenuation can be used to characterize tissue properties of the human breast. Both quantitative ultrasound (QUS) and ultrasound tomography (USCT) can provide attenuation estimation. However, limitations have been identified for both approaches. In QUS, the generation of attenuation maps involves separating the whole image into different data blocks. The optimal size of the data block is around 15–30 pulse lengths, which dramatically decreases the spatial resolution for attenuation imaging. In USCT, the attenuation is often estimated with a full wave inversion (FWI) method, which is affected by background noise. To achieve a high-resolution attenuation image with low variance, a deep learning (DL)-based method was proposed. In the approach, RF data from 60 angle views from the QTI Breast Acoustic CT (BACT) scanner were acquired as the input and attenuation images as the output. To improve image quality for the DL method, the spatial correlation between speed of sound (SOS) and attenuation were used as a constraint in the model. The results indicated that by including the SOS structural information, the performance of the model was improved. With a higher spatial resolution attenuation image, further segmentation of the breast can be achieved. The structural information and actual attenuation values provided by DL-generated attenuation images were validated with the values from the literature and the SOS-based segmentation map. The information provided by DL-generated attenuation images can be used as an additional biomarker for breast cancer diagnosis.

## Graphical Abstract

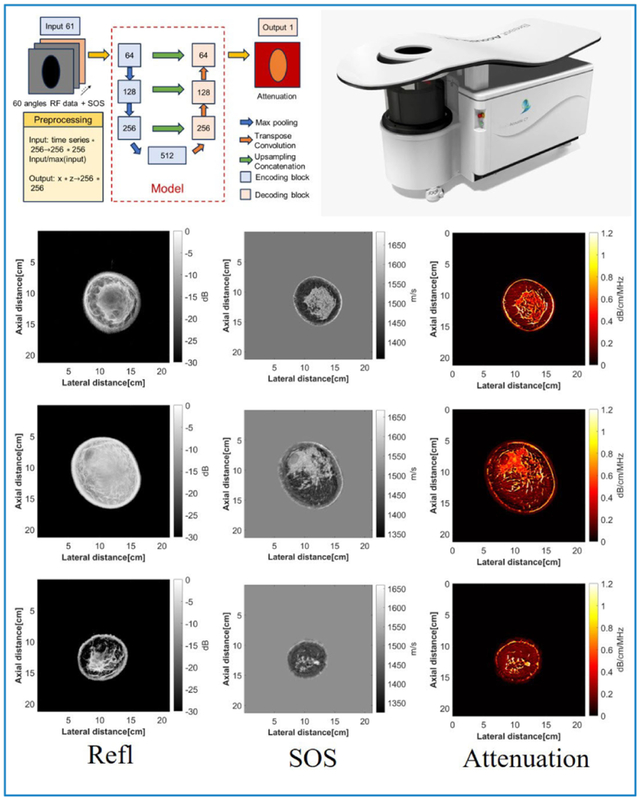

## I. Introduction

Breast cancer is one of the primary causes of death among women [1]. The importance of early detection and continuous monitoring cannot be overstated, as they are essential to improving survival rates. Given its high impact on women’s health, there is a pressing need for effective diagnostic tools and monitoring methods. Ultrasonic attenuation is a fundamental property of tissue and can be related tissue state, including the potential to diagnose disease [2], [3], [4] and to monitor therapy [5], [6]. Several studies have demonstrated that combining tissue speed of sound (SOS) and attenuation characteristics could provide a better diagnosis of breast cancer [7], [8]. For instance, low attenuation in the human breast lesions can indicate fatty tissue or medullary carcinoma, and high attenuation lesions can indicate an infiltrating ductal carcinoma or fibrosis [9].

There are two main ways to create ultrasonic attenuation maps, either using quantitative ultrasound (QUS) or using ultrasound tomography (USCT), e.g., the QTI Breast Acoustic CT^1^ (BACT) scanner [10], [11], [12], [13]. QUS is an imaging technique, which includes spectral-based parameterization based on the backscattered RF or IQ signal [14], [15]. There are many spectral-based methods, including spectral difference method, spectral log difference (SLD) method, and hybrid method. In studies comparing the methods, the SLD method was found to have the best performance within heterogeneous tissues [16]. Other previous studies have shown that high-accuracy attenuation maps with low variance could be acquired with a piecewise homogeneous region [17], [18]. However, there is a tradeoff between the spatial resolution and variance of attenuation estimation because QUS methods involve separating the whole image into different data blocks. When the data blocks are larger, the spatial resolution will decrease but the variance will decrease assuming uniform scattering statistics within the data block, and vice versa. Therefore, improving the tradeoff between attenuation estimate variance and attenuation image spatial resolution has been an important avenue of investigation. Attempts to expand this tradeoff include using regularization techniques [17], [18] or full angular spatial compounding (FASC) [19], [20]. However, even with regularization, the QUS-attenuation estimation methods cannot deal with fine structures because the optimal block size is still around 15–30 pulse lengths, which will decrease the spatial resolution of the original signal by more than 15 times. For attenuation imaging, most approaches focus on estimating values within a homogeneous tissue such as liver, or a region of interest where attenuation characteristics are of significance irrespective of the underlying structures.

For applying FASC, data from an ultrasound tomography scanner are required. Previously, we implemented the SLD method on a tomography imaging system to generate attenuation images [21]. The ability to do FASC can result in improved attenuation images in terms of both variance and spatial resolution. The corresponding SOS images were used to correct for refraction, and the subsequent SLD attenuation images were compared with attenuation images constructed using full wave inversion (FWI). However, the SLD images combined with FASC still provided images with low spatial resolution.

Deep learning (DL) in medical imaging has received much attention in recent years, including ultrasonic imaging. Many studies have investigated DL for image translation tasks, including SOS estimation [22], [23], super-resolution power Doppler imaging [24], [25], etc. Example architectures include using a UNET structure with convolutional neural networks (CNNs) [26] or generative adversarial network (GAN) [27] for image translation tasks. There are two previous studies that used DL for attenuation estimation [28], [29]. Both the works used a UNET CNN structure and trained the neural networks with some simulation data [30], [31]. However, there are two main drawbacks of these two studies. The first is that the input data were simulated pulse-echo data, which are either the spectrum of RF data or enveloped data, which resulted in reduced information in the raw RF or IQ data. The second is that the test dataset was either a homogeneous phantom or a homogeneous human tissue, such as liver, which were not objects with fine underlying structures.

In this work, we make use of a public dataset having simulated breasts with fine structures [32], which provides the ground-truth sound speed, attenuation, and density maps of different kinds of breasts. In addition, we have access to human breast data from a QTI BACT system that can provide 60 angles of reflection mode IQ data and accurate sound speed reconstruction [33]. Using these data, we train a model to provide attenuation images with fine structures to assist in segmentation of breast tissues. This study provides two main contributions. The first is that 60-angle IQ reflection mode data along with the sound speed images are used as the input for training the model. Because of the Kramers–Kronig relationships [34], the SOS estimation is used as a guide to generate a more reasonable attenuation imaging model. Specifically, the structure of the breast in terms of SOS is assumed to be similar in terms of structures having different attenuation values. Because the QTI tomography system can generate high-resolution SOS images, which is assumed to be close to the ground truth, then predicting the attenuation using the model, the “ground-truth” SOS images can be used as the input. The second is that by training the model on simulations of breast tissues with fine structure, our model can produce attenuation imaging that includes fine structures. As a result, this DL-based method can produce much higher spatial resolution attenuation images compared with QUS-based methods.

## II. Methods

### A. Attenuation Imaging

#### 1) FWI Method:

The attenuation imaging for USCT is implemented with the FWI method. It essentially solves a non-linear inverse problem. Starting with the Helmholtz equation

(1) $$\nabla^{2}p(x)+k^{2}(x)p(x)=0$$

where p(x) denotes the pressure field, and k(x) denotes the wavenumber, which is defined as k(x)=(ω/c(x))-jα, i.e., the real part of the wavenumber denotes the sound speed and the imaginary part denotes the attenuation. The object function for the inverse problem is defined as

(2) $$\gamma \left(x\right)=\frac{k}{k_{0}}-1=\frac{c_{0}}{c\left(x\right)}-1-j\frac{\alpha c_{0}}{2\pi f}.$$

Using the paraxial approximation and minimizing the object function iteratively, the sound speed and the attenuation can be inferred. In short, the FWI method for attenuation imaging can be viewed as a mapping from the measured wave fields (RF data) to sound speed c and attenuation α

(3) $$\text{FWI}:\;\text{RF}\overset{ℱ}{\to }(c,\alpha )\text{.}$$

#### 2) SLD Method:

The attenuation imaging for QUS based on the backscatter was usually implemented with the SLD method. It is based on modeling the spectrum of the received RF data using a sliding window. Essentially, it solves the equation

(4) $$\text{ln}\left(\frac{S_{s}\left(f,z_{p}\right)}{S_{s}\left(f,z_{d}\right)}\right)-\text{ln}\left(\frac{D_{s}\left(f,z_{p}\right)}{D_{s}\left(f,z_{d}\right)}\right)=4\left(z_{d}-z_{p}\right)\alpha_{s}\left(f\right)+\text{const}$$

where $\text{ln}\;\left(\left(S_{s}\left(f,z_{p}\right)/S_{s}\left(f,z_{d}\right)\right)\right)$ denotes the spectrum for the two windows of the backscattered RF data, $z_{d}-z_{p}$ is the distance between the center of two windows, $\text{ln}\;\left(\left(D_{s}\left(f,z_{p}\right)/D_{s}\left(f,z_{d}\right)\right)\right)$ denotes the diffraction term, and the constant is related to backscatter coefficient (BSC). In short, the attenuation is estimated by fitting a line over an analysis bandwidth for (4), which can be viewed as a mapping from the backscattered RF data to BSC and attenuation

(5) $$\text{SLD}:\;\text{RF}\overset{ℳ}{\to }\left(\text{BSC},\alpha \right).$$

### B. Dataset

#### 1) Simulation Dataset:

**Fig. 1.**
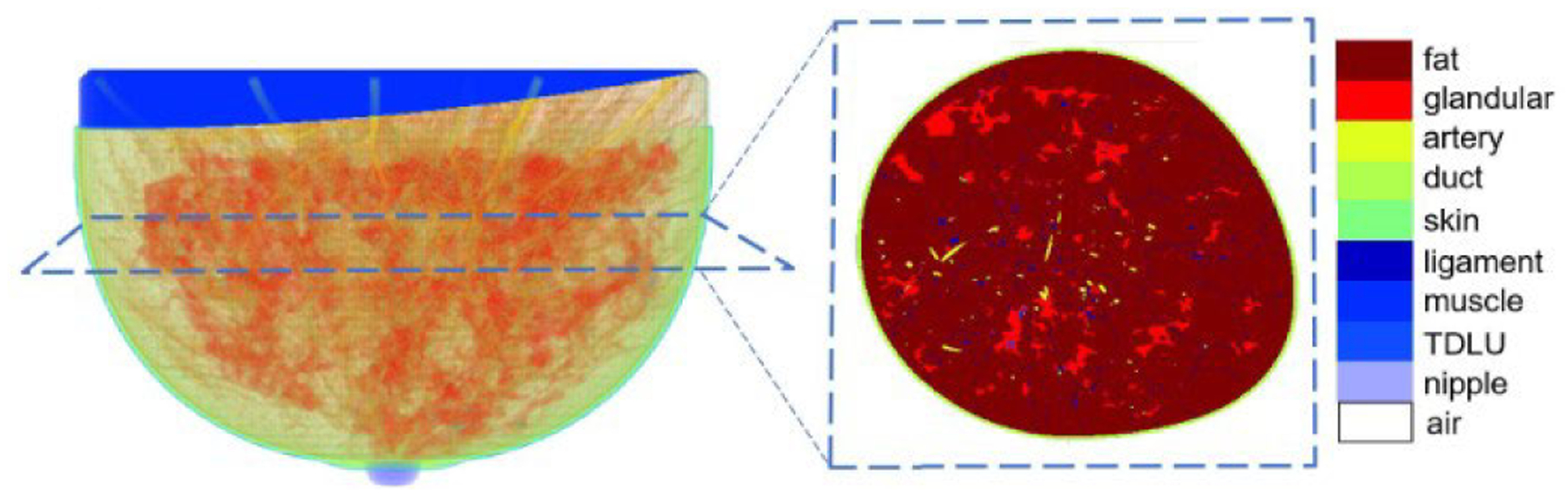
Schematic of the 3-D digital breast phantom in the dataset [32].

To generate a dataset for training the model, the public dataset [32] which was generated by the VICTRE software [35] was used. The digital breast phantoms in the dataset have 3-D structures with different regions of tissues; an example is shown in Fig. 1. There are four different kinds of breast phantoms in the dataset, and these four kinds of breast have four different levels of breast density.

1. Breast A is almost entirely fat.
2. Breast B has scattered areas of fibroglandular density.
3. Breast C is heterogeneously dense.
4. Breast D is extremely dense.

**Fig. 2.**
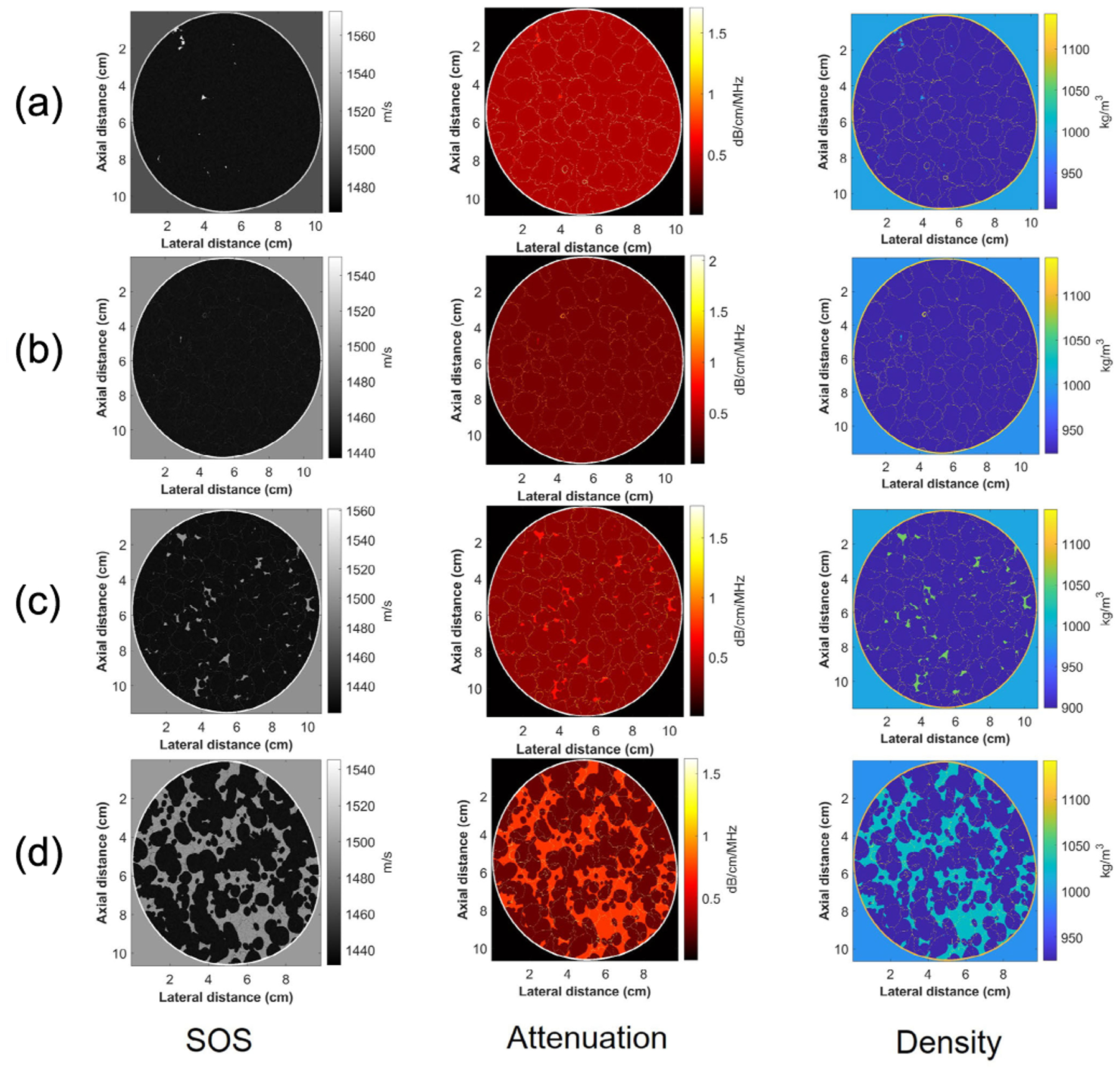
Example images of a 2-D slice (from slice 117th) of the 3-D digital breast phantoms from the dataset. (a) Breast A. (b) Breast B. (c) Breast C. (d) Breast D.

In Fig. 2, example images of these four different breasts are displayed. Each of the images is a 2-D slice within the 3-D dataset. The sound speed (SOS), attenuation and density images are shown. The k-wave [30] was used to generate the 60-angle backscattered RF signals from each slice based on the array characteristics of the QTI BACT scanner. The 2-D SOS, attenuation, and density slices in the dataset were input into the k-wave simulation. The details of simulation parameters can be found in Table I. All the simulation settings were similar to the QTI ultrasound tomography system except that the simulation required more element numbers to cover the whole image field of view. The pipeline of the dataset generation can be found in Fig. 3, and the total number of RF signal for each breast can be found in Table II. All the simulations were processed with a GPU NVIDIA RTX A5000, taking around one hour to produce one slice of 60-angle RF signals.

#### 2) Phantom Dataset:

**Fig. 4.**
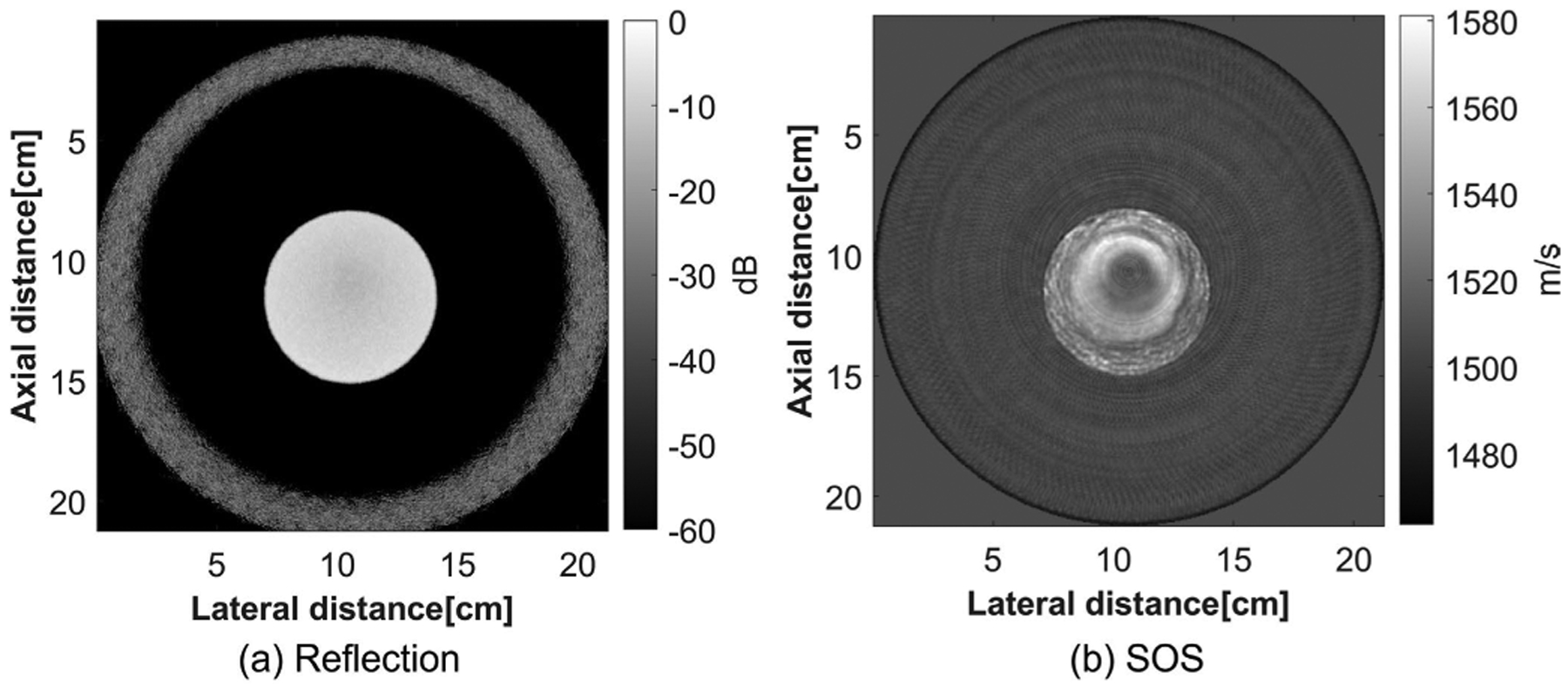
Images of the reference phantom that were scanned. (a) Reflection image of the homogeneous phantom. (b) SOS image of the homogeneous phantom.

**Fig. 5.**
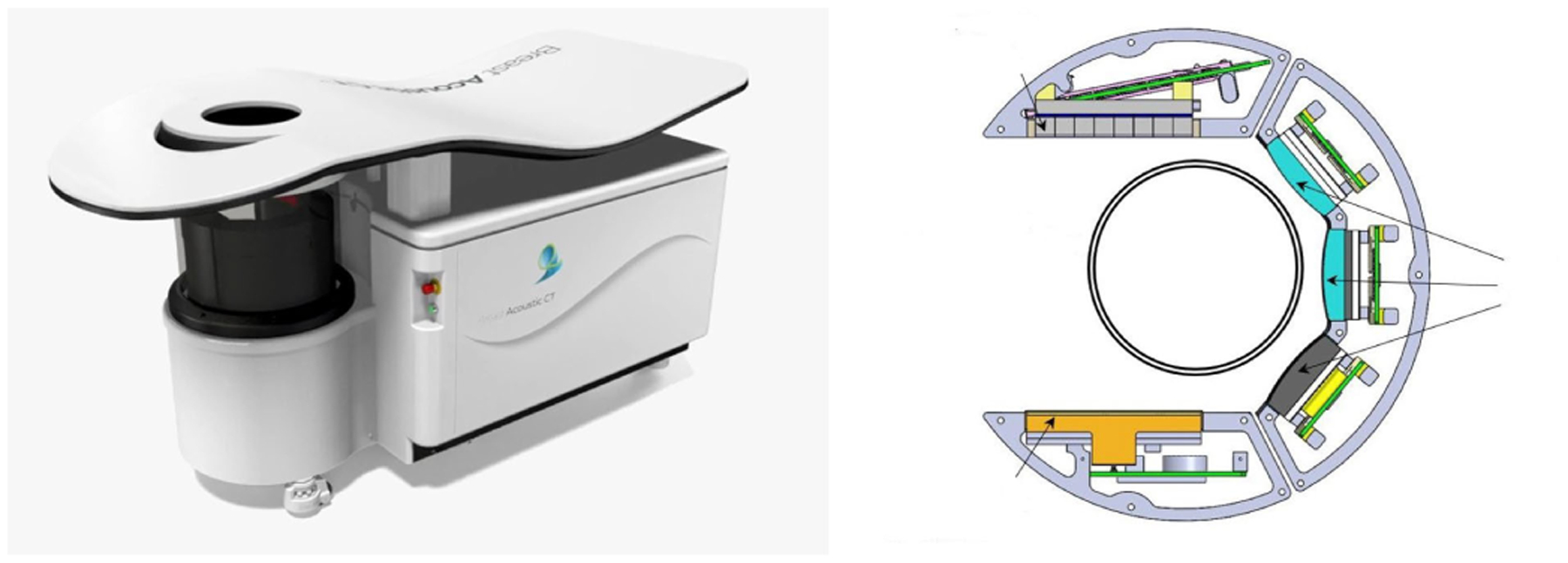
(Left) QTI BACT scanner and (right) transmitter (orange) and receiver array (light gray) for transmission and three transceivers (blue and dark gray) on the right side of the array holder for obtaining reflection mode data [33], [38].

Four datasets from tissue mimicking phantoms and human breasts scanned by QT Imaging Holdings, Inc., Novato, CA, USA, were acquired. The QTI ultrasound tomography system can generate both sound speed and attenuation images using the FWI method and 60-angle reflection images. The details of this imaging system can be found in previous works [33], [36]. One phantom was constructed for scanning in the QTI BACT scanner. It was a homogeneous phantom made with 0.03-g/mL agar, 0.1-g/mL glass beads, and 0.05-g/mL graphite randomly distributed spatially. This phantom was also used as the reference phantom for the QUS method. The insertion loss method was used [37] to estimate the attenuation of the phantom. The attenuation of the phantom was estimated as 0.76 dB/cm/MHz. The SOS and reflection mode images of the phantom are shown in Fig. 4. The system is shown in Fig. 5.

#### 3) In Vivo Dataset:

In vivo breast data were acquired by scanning patients with the QTI BACT scanner (QT Imaging Holdings, Inc.). The study was approved by the Institutional Review Board of Western Institutional Review Board (protocol code BR004; WIRB Pro Num: 1167638; date of approval: August 25, 2017). The first case is a normal breast and the second case is a breast with a cyst region. The third case is a breast with cancer. In addition, nine patients with breast cancer were scanned at the Sunnybrook Health Sciences Centre, Toronto, ON, Canada (protocol code 5552).

### C. Neural Network Architecture

The UNET structure CNN was adopted in this study. The encoder consists of a max-pooling layer followed by a double convolution block, which includes repeated convolution layers, batch normalization, and ReLU activation. The decoder features transpose convolution layers and the same double convolution blocks. However, instead of one channel input, 61 channels were used as input in this study, i.e., the network can be viewed as a mapping from 60-angle RF data and SOS to the attenuation, which is

(6) $$\text{DL}:\;\left(d_{\text{RF}},c\right)\overset{𝒢}{\to }\alpha .$$

**Fig. 6.**
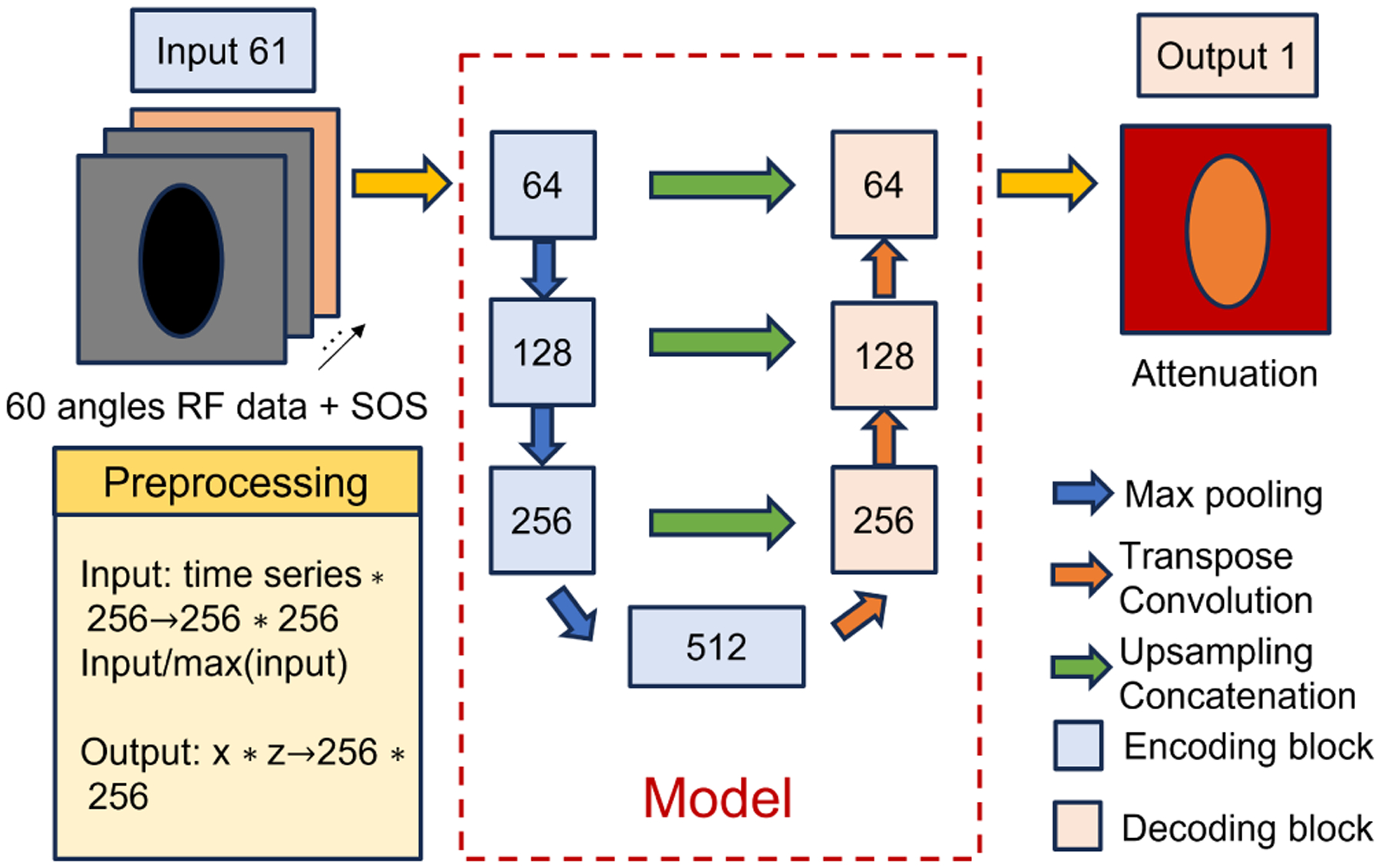
Structure of neural networks.

Here, $d_{\text{RF}}$ represents the 60-angle RF data with dimensions 60×H×W, and c denotes the one-channel SOS map with dimensions 1×H×W. The DL model 𝒢 learns a mapping from the combined input ($d_{\text{RF}},c$) to the attenuation map $\alpha \in R^{1\times H\times W}$. An ablation study, as described later, was conducted to explain the effectiveness of adding the sound speed as an input. Due to the limit of 24-GB GPU storage (NVDIA TITAN RTX), both the input and output images were resized to 256 × 256, and only the slices between the 200th to 300th in the simulation breast dataset were used. The loss function was SSIM with an $L_{2}$-norm regularization term to avoid overfitting, which is defined as

(7) $$\text{Loss}=\frac{1-\text{SSIM}\left(x,y\right)}{2}+\lambda \underset{k}{\sum }{\left\|\theta_{k}\right\|}^{2}$$

where $\theta_{k}$ denotes the model parameters, λ=0.001 in all the experiments, and the SSIM will be defined later in the image quality metrics. The Adam optimizer with a learning rate of 0.001 and a batch size of 32 and 300 epochs were used in the training process. The structure of the neural networks and the dataset composition are found in Fig. 6 and Table III.

### D. Ablation Study

To demonstrate the effectiveness of adding the sound speed image as an input, an ablation study was conducted. Five cases were tested, which are as follows.

1. Envelope-detected + SOS.
2. RF + SOS.
3. Envelope-detected only.
4. RF only.
5. SOS only.

The ablation study was conducted on the test set of the simulation breast phantom.

### E. Image Quality Metrics

To quantify the similarities and differences between different methods, three metrics were chosen. The first metric was structural similarity index measure (SSIM) [39] and is defined as

(8) $$\text{SSIM}(x,y)=\frac{\left(2\mu_{x}\mu_{y}+C_{1}\right)\left(2\sigma_{xy}+C_{2}\right)}{\left(\mu_{x}^{2}+\mu_{y}^{2}+C_{1}\right)\left(\sigma_{x}^{2}+\sigma_{y}^{2}+C_{2}\right)}$$

where $\mu_{x},\mu_{y},\sigma_{x},\sigma_{y}$, and $\sigma_{xy}$ are the local means, standard deviations, and cross-covariance for images x and y. $C_{1}$ and $C_{2}$ are two regularization constants, where $C_{1}=(0.01\times L{)}^{2}$ and $C_{2}=(0.03\times L{)}^{2}$, L stands for the dynamic range of the pixel values. In this study, the dynamic range for attenuation values was considered between 0 and 3, so L=3 was set for all the experiments calculation. SSIM results in a value between 0 and 1. The closer to 1, the higher the similarity between two images, and the higher the image quality.

The second metric used to quantify performance was the root-mean-square error (RMSE). The RMSE is defined as

(9) $$E=\sqrt{\frac{1}{n}\sum_{i=1}^{n}{\left|A_{i}-F_{i}\right|}^{2}}$$

where F is the forecast matrix and A is the actual matrix made up of n observations. The lower the RMSE, the closer the forecast matrix is to the actual matrix.

The third metric used to quantify the differences between segmentation maps was the intersection over union (IoU). The IoU is defined as

(10) $$\text{IoU}(A,B)=\frac{\left|A\cap B\right|}{\left|A\cup B\right|}$$

where A and B are two segmentation maps with either 0 or 1 value.

## III. Results

### A. Simulation Results

**Fig. 7.**
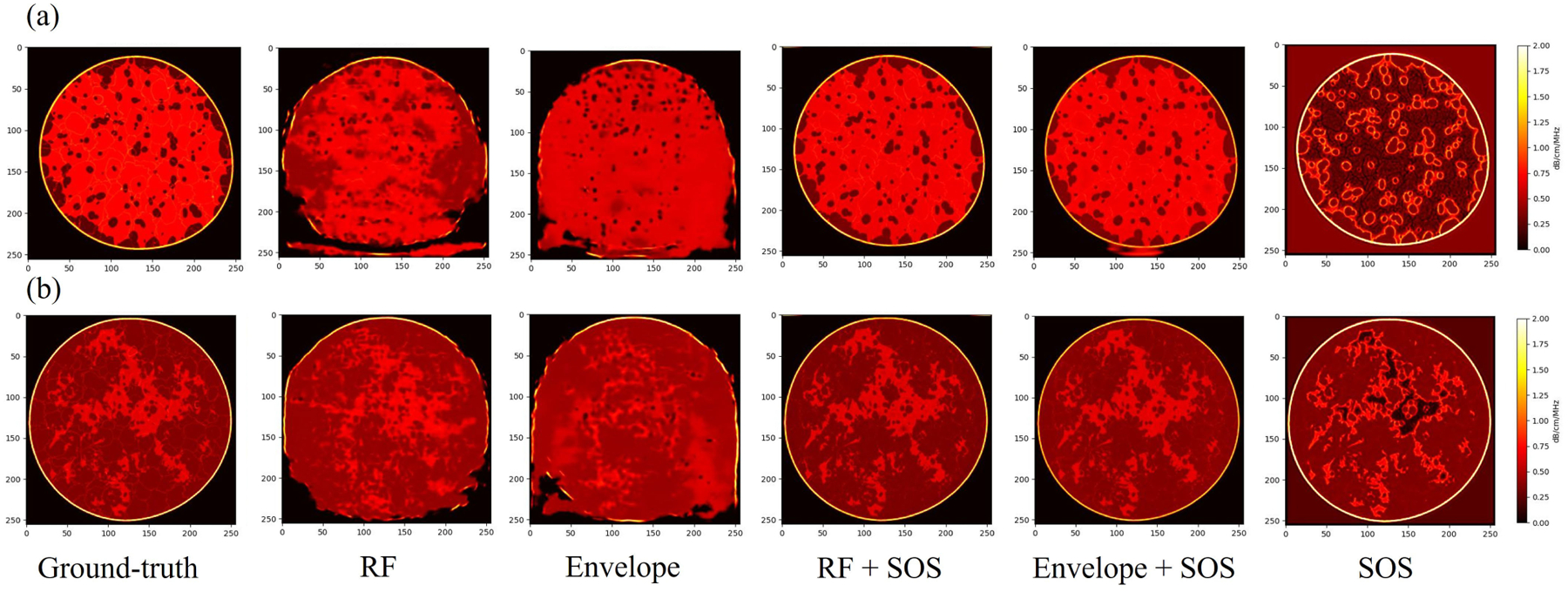
Result of two cases in the test set of A + B + D. (a) and (b) are two different slices.

**Fig. 8.**
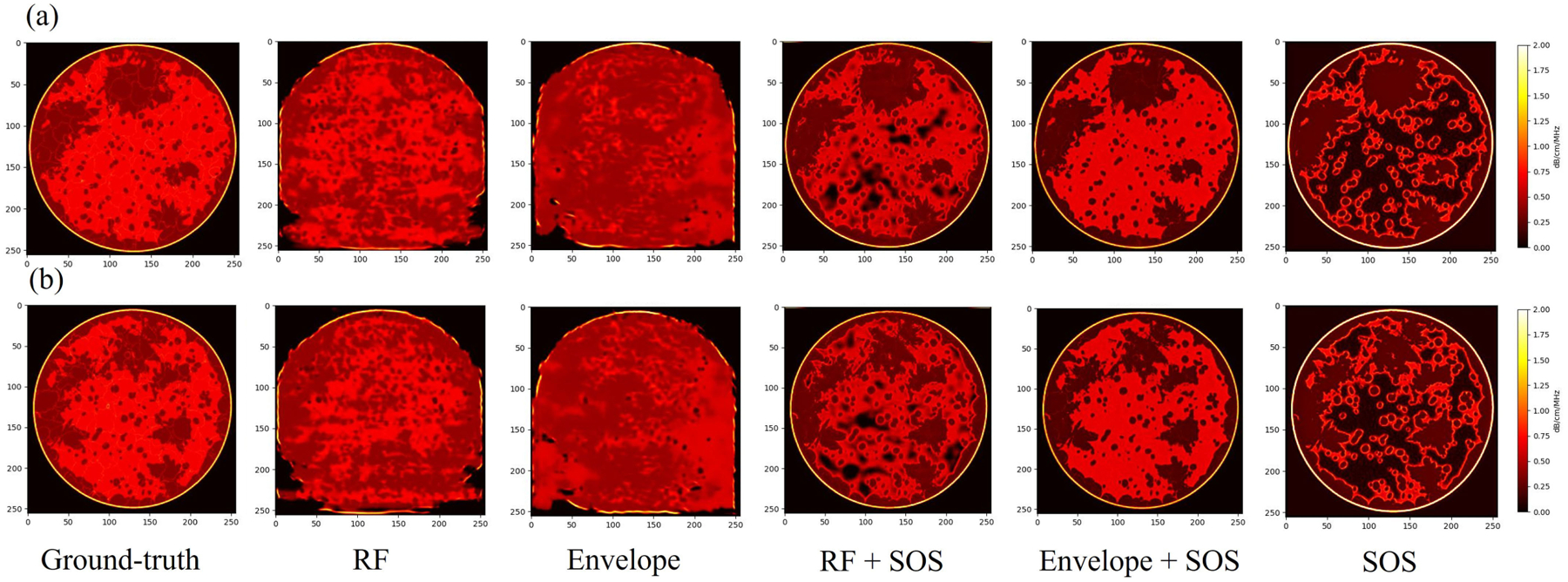
Result of two cases in the test set of C. (a) and (b) are two different slices.

The results from the simulated breast phantoms are shown in Figs. 7 and 8. For both the split test set of breast phantoms A, B, and D, and the unseen test set breast phantom C, five different cases along with the ground-truth attenuation images are shown. In addition, for quantitative analysis, the SSIM and RMSE between five different cases and the ground-truth were calculated and are listed in Table IV and V. From the results, first, the Envelope + SOS case provided the best performance, with the lowest RMSE and highest SSIM among all five cases. The attenuation images from Envelope + SOS case reflected the fine structures in the breast phantoms. In addition, the result indicates that when including the SOS image into the input, the generated model resulted in a marked improvement compared with the predicted attenuation images without using sound speed. However, only using the SOS image as the input did not achieve good performance, which illustrates that the SOS map should be used as an extra input in addition to the 60-angle RF signal.

**Fig. 9.**
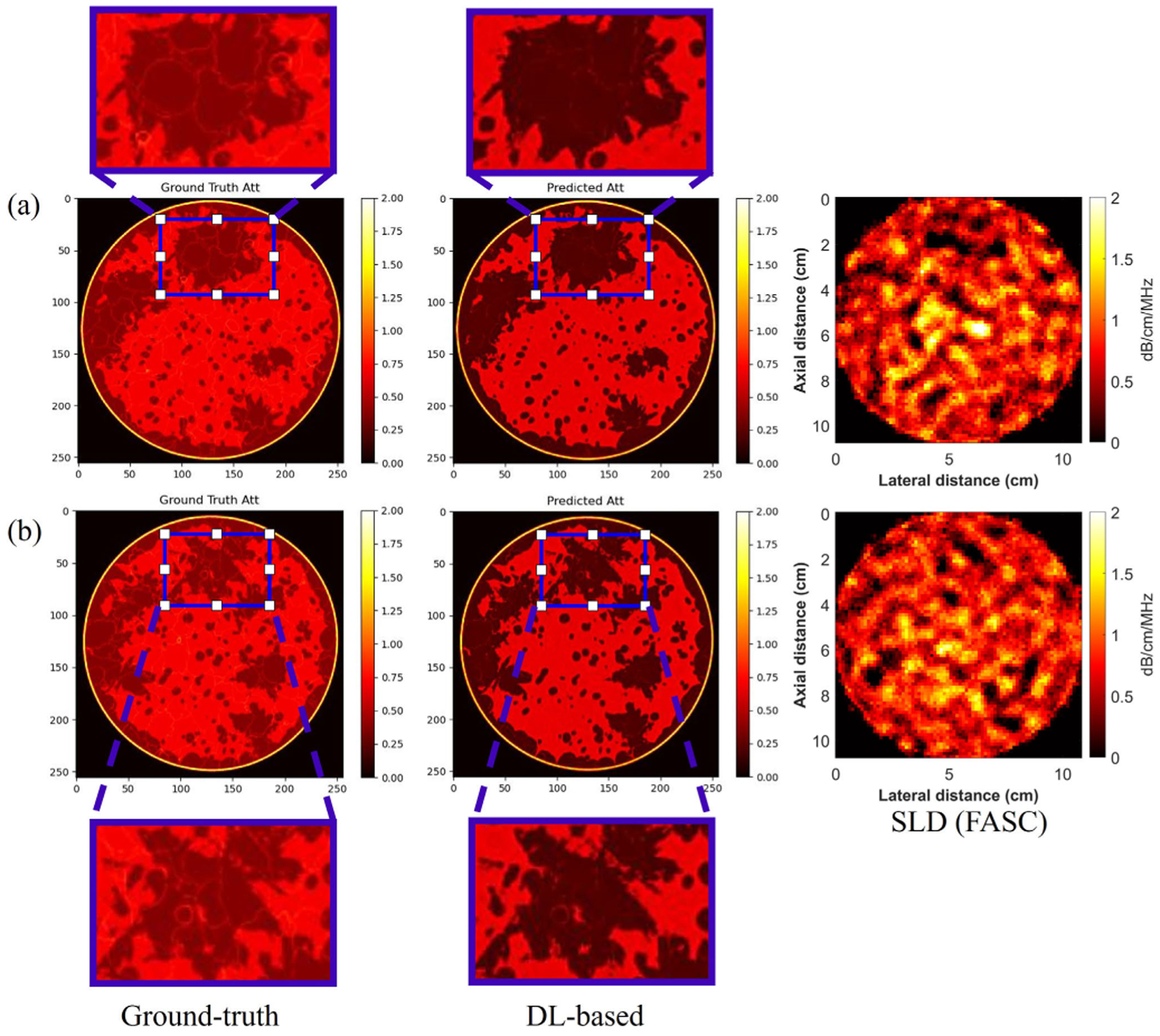
Attenuation images generated using the DL method in comparison to the SLD method. (a) and (b) Correspond to two different breast realizations.

To compare the DL-based method with the SLD method, the unseen breast phantom C was analyzed, and 60-angle spatial compounding was used with the SLD method. From Fig. 9, the DL-based method resulted in a higher spatial resolution attenuation image with relatively finer structures visible, whereas the SLD method resulted in a lower spatial resolution image of the underlying structure. Moreover, ROIs of the ground truth and the DL-based method were chosen. However, the thin lines apparent in the zoomed in ground-truth attenuation images are missing in the DL-based images, which illustrates that there are some resolution limits with the DL-based method.

### B. Physical Phantom Results

Drawing from the simulation results, the model generated with Envelope + SOS provided the best performance. As a result, this model was used for processing the physical phantoms and in vivo breast data. One homogeneous phantom was scanned in the QTI Imaging system, and the attenuation image was reconstructed using the DL-based method, the SLD method, and the FWI method.

**Fig. 10.**
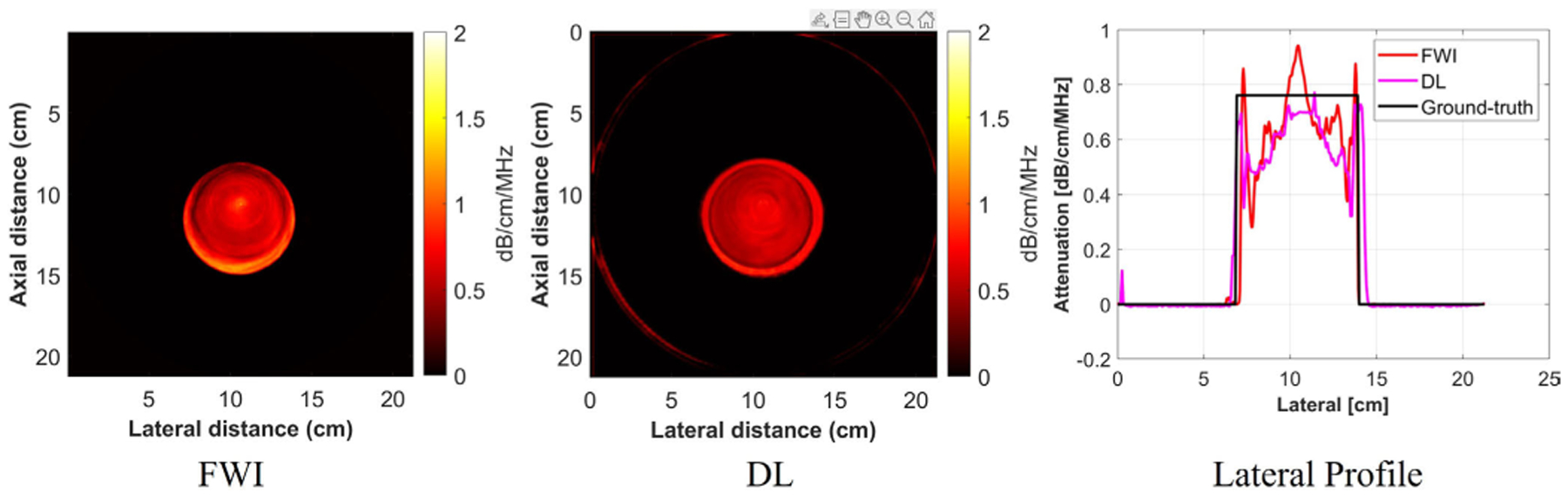
Attenuation images of the homogeneous phantom using different reconstruction techniques.

Because the homogeneous phantom was used as the reference phantom for SLD method for diffraction correction, the resulting attenuation image using the SLD method for this phantom is not available. As a result, the DL method and FWI method were compared for the homogeneous phantom, and the results are provided in Fig. 10 and Table VI. From the results, the estimated mean attenuation value for the DL method was 0.69, which is close to the ground-truth attenuation value. Compared with the FWI method, the DL method was closer to the ground-truth value with a lower variance. The lateral profile was also plotted. From the lateral profile, the DL method had a lower variance with sharper edges compared with the FWI method.

### C. Test Dataset of In Vivo Breast

**Fig. 11.**
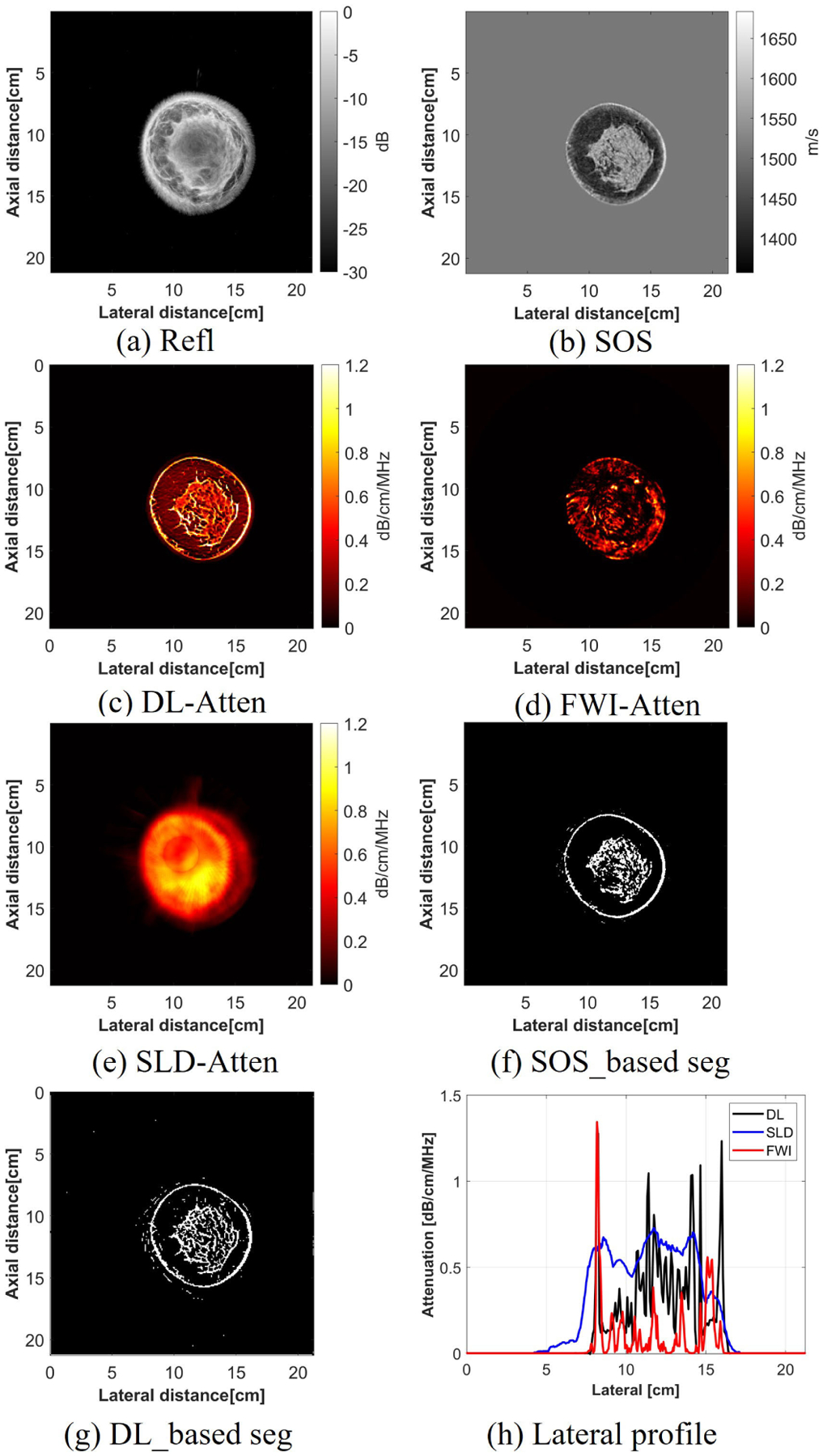
Attenuation imaging results for the normal breast using different reconstruction methods.

**Fig. 12.**
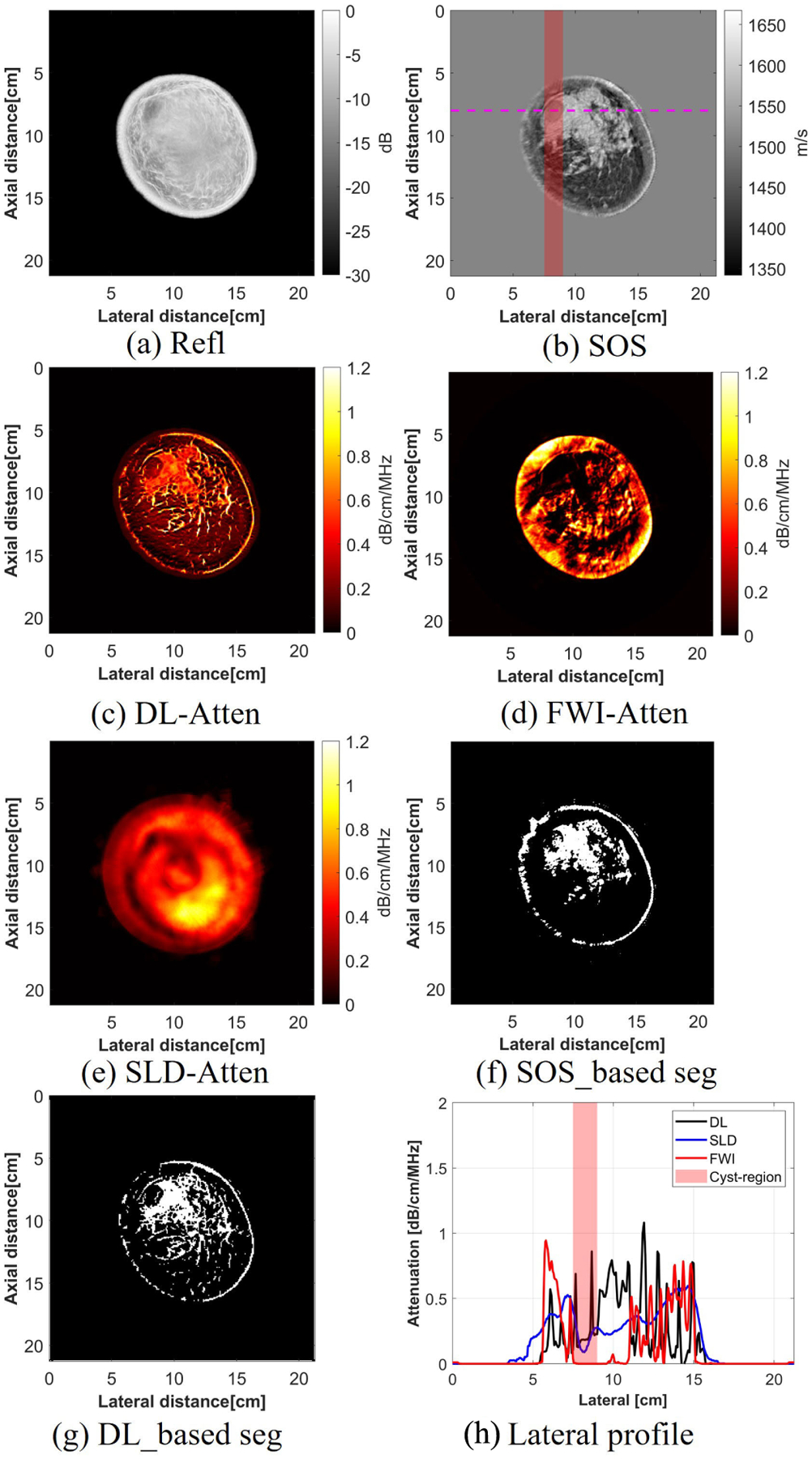
Attenuation imaging results for the breast with a cyst using different reconstruction methods.

**Fig. 13.**
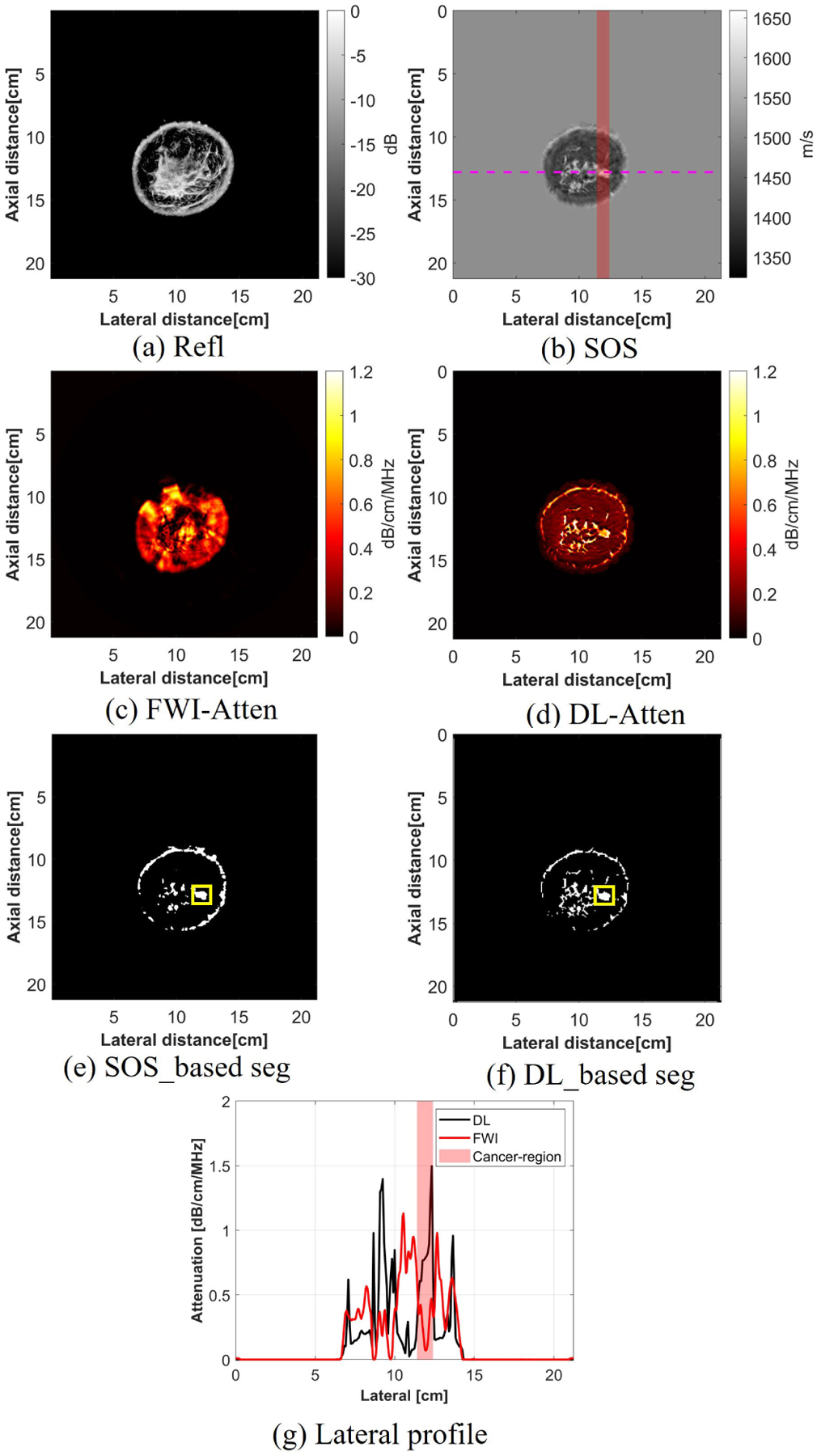
Attenuation imaging results for the breast with cancer using different reconstruction methods.

Three female breasts were scanned in vivo and processed with the DL method, the SLD method, and the FWI method. The first was a normal breast (Fig. 11), the second was a breast with a cyst (Fig. 12), and the third was a breast with cancer (Fig. 13). For the normal breast, after getting the attenuation map based on the DL method, a threshold was applied on both the SOS map and the attenuation map, and two segmentation maps were obtained. The DL-based segmentation map with respect to the SOS-based segmentation map was similar, with a correlation coefficient of 0.67, which illustrates the ability of DL-based method to capture the fine structures in the breast tissue. It also indicates the accuracy of the DL-based attenuation map, which aligns well with the SOS segmentation result. However, the SLD method could not construct high spatial resolution images and failed to achieve the segmentation based on the attenuation map. This could also be observed in the lateral profile, where the profile of the SLD image is smooth while the DL profile has sharp edges that appear to capture the small changes in attenuation.

For the breast with a cyst, the same threshold was applied, and the ground-truth cyst region was denoted by SOS map. The lateral profiles of the images reconstructed using the SLD, DL, and FWI methods were plotted, and the cyst region was denoted in a red rectangle. From the lateral profile, the DL method was able to localize the cyst region correctly with sharp edges. In comparison, the SLD method could also get the correct size estimate of the cyst region, but with smooth edges due to low spatial resolution attenuation maps. The FWI method was able to get a sharp edge, but overestimated the size. From the segmentation maps generated with both the SOS and DL-based attenuation images, the high correlation (0.71) between the two indicates the successful capture of both the cyst region and other fine structures in the breast. In addition, the IoU of the cyst region was calculated, and the high IoU (0.73) indicates that the DL captured the cyst region successfully.

For the breast with cancer, after applying the threshold, the cancer region was denoted in yellow rectangles. From both the SOS and DL-based attenuation maps, the high SOS corresponded to the cancerous region and this corresponded to a high attenuation region of the same size and location in the breast. However, this cancer region could not be captured with the FWI method. From the segmentation maps from both the SOS and DL-generated attenuation images, a correlation coefficient of 0.64 and an IoU of 0.93 of the cancer region illustrate the high correlation between two segmentation maps, and the successful capture of the cancer region demonstrates the ability of using DL-generated attenuation maps to characterize breast tissues.

**Fig. 14.**
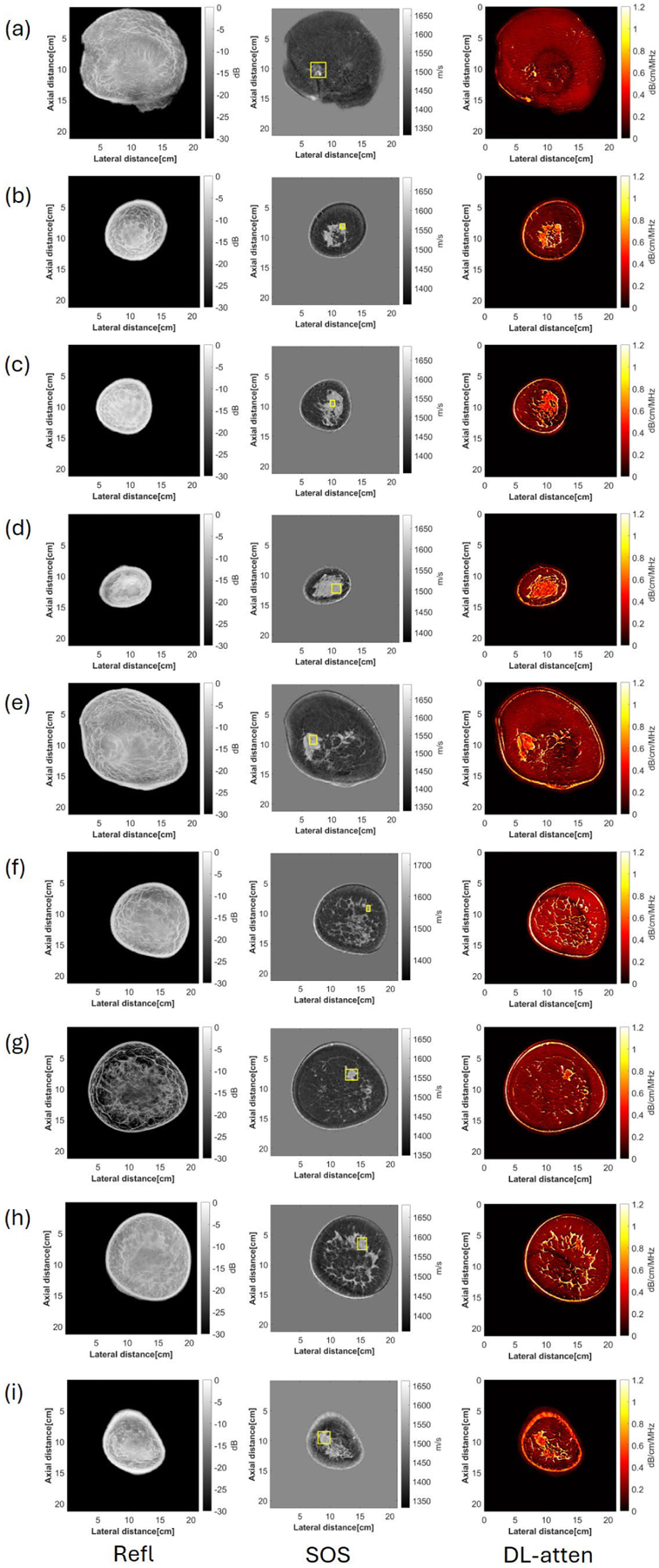
Reflection, sound speed, and DL-based attenuation images of additional nine cancer cases. (a)-(i) Represent nine different cancer cases.

In addition, nine patients with breast cancer were scanned and data were processed with the DL method, and the reflection, SOS, and DL-based attenuation images can be found in Fig. 14. The pathology types of these nine cancers include infiltrating duct carcinoma (IDC), infiltrating lob-ulillar carcinoma (ILC), metaplasic carcinoma (MetCa), and mucinous carcinoma (MucCa), and the cancer regions were denoted with a yellow box on the SOS image. The mean and variance of the attenuation values were calculated for each case (see Table VII), and the mean value of all the nine cases was 0.59 dB/cm/MHz, which aligns well with the previous literature [40], which was 0.57 ± 0.14 dB · cm^−1^ ⋅ MHz^−1.3^.

## IV. Discussion

Both QUS and the QTI BACT scanner can generate ultrasound attenuation images. In QUS, the attenuation images are generated with reflection mode images. In the QTI BACT scanner, the attenuation images are generated with transmission mode images. However, both these approaches face challenges when generating attenuation images. In QUS, there is a tradeoff between spatial resolution and estimate variance. Typically, even with techniques such as regularization or FASC, the spatial resolution of attenuation images is reduced by up to 15 times compared with the resolution cell of the scanner. In the QTI BACT scanner, the FWI method can be used to reconstruct attenuation images with the paraxial approximation. However, because the attenuation is encoded in the imaginary part of the wavenumber, the attenuation images are of much lower quality than the sound speed images. Recall that the inversion problem for the imaginary part of the wavenumber is numerically poorly conditioned compared with the real part [41].

Recently, DL has been used widely in medical imaging and ultrasound, and it can be used to do many image translation tasks. Based on the success of DL in medical imaging, a DL-based method was adopted in this study to achieve a high spatial resolution attenuation image with low variance. In many image translation tasks, a UNET is used. However, most of the studies used one-channel RF or IQ data as the input to train the neural network. In this study, 60-angle of envelope-detected data combined with a registered SOS image were used as the input. To understand the contributions of the SOS images in the DL reconstructions of attenuation, an ablation study was conducted, and five different cases were tested. The results demonstrated that the performance of the neural network improved dramatically when adding the sound speed image into the input channels, and the 60-angle of envelope-detected data combined with the registered SOS image provided the best performance.

### A. Sensitivity Analysis

In theory, the relationship between SOS and attenuation is a causality-imposed Kramers–Kronig relationship. In soft tissues, the attenuation is usually assumed to be linear with respect to frequency, i.e., the exponent term in the power law is 1. Deriving from the Kramers–Kronig relationship under the exponent term equal to 1 [42], we have

(11) $$\alpha_{0}^{\prime }=\alpha_{0}\left(1-\frac{2\Delta }{c_{g}}\right)-\frac{2\Delta }{\pi }\alpha_{0}^{2}$$

where Δ denotes the change in SOS, $c_{g}$ is the original SOS, $\alpha_{0}$ is the original attenuation in unit Np/m/Hz, and $\alpha_{0}^{'}$ is the new attenuation in unit Np/m/Hz. The derivation details can be found in the Appendix.

Empirically, in soft tissues, the group velocity is around 1540 m/s, and the attenuation coefficient slope is around 0.75 dB/cm/MHz. Substituting the numbers in (11), supposing a perturbation of 10–1550 m/s, the perturbed calculated attenuation coefficient slope will be 0.74 dB/cm/MHz. As a result, the 10-m/s change in SOS will lead to a ~0.01-dB/cm/MHz change in attenuation, which is negligible.

**Fig. 15.**
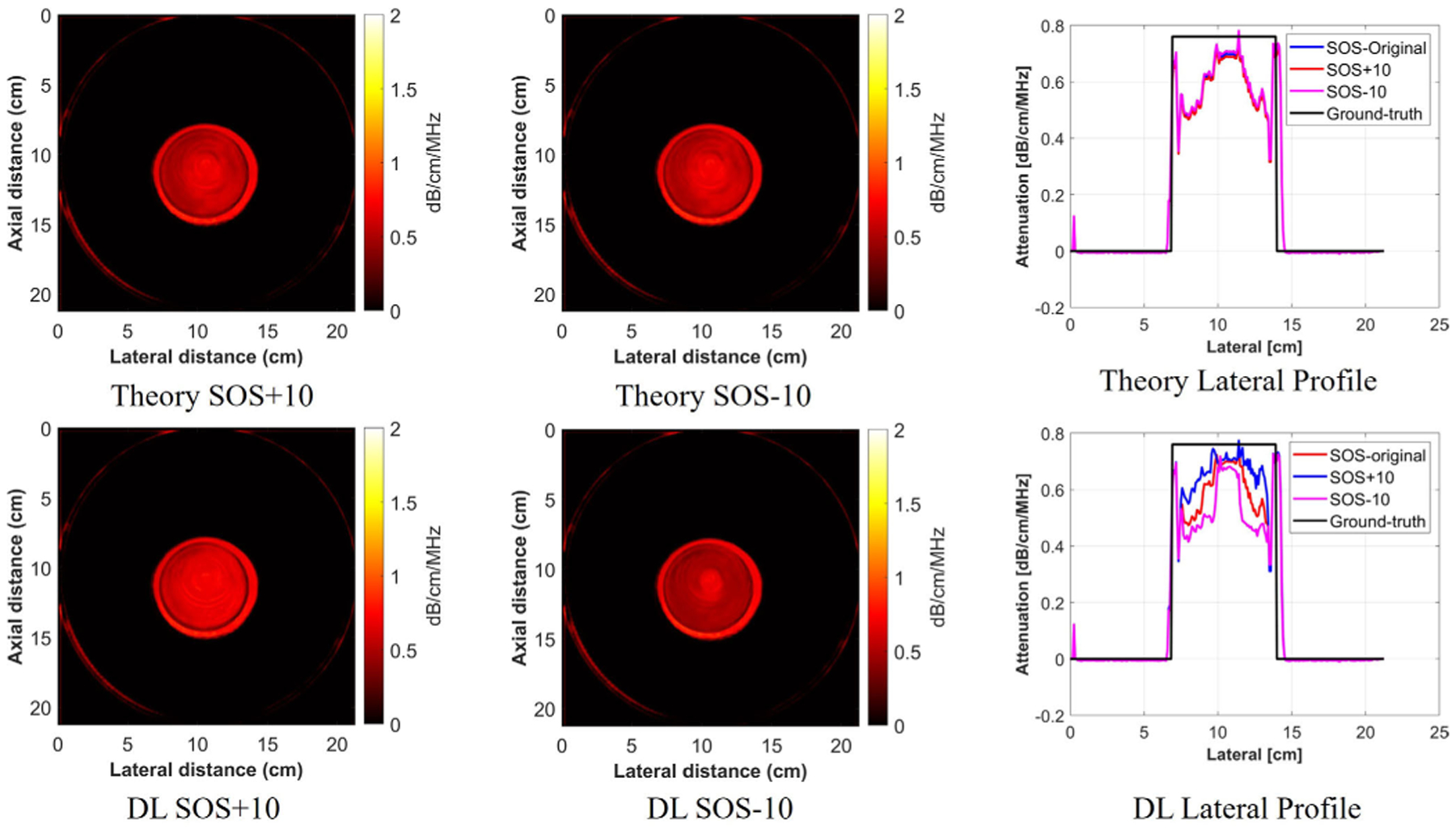
Lateral profile attenuation plot of the theory and DL with different SOS input values.

In this study, the neural network required the SOS map for best performance. In simulation, the SOS map was perfectly known, but in the test dataset, the SOS map was reconstructed with FWI method, which means the true value was not perfectly known. However, considering the high resolution and high SNR of the SOS map that QTI was able to produce, the SOS map from QTI was labeled ground truth in the test dataset. Furthermore, the SOS images were used as a priori information to help predict the attenuation images, i.e., it is the structure information provided by SOS images that correlate to the structure of attenuation images, but not the actual values of SOS. To test this assumption, an experiment on the homogeneous phantom was conducted. A mask was applied on the phantom region, and the SOS values of the phantom region were changed by ±10 m/s. The image and the lateral profile of the phantom are shown in Fig. 15, with both in theory deriving from (11) and from DL. The SSIM and RMSE of the predicted attenuation values between the using original SOS and ±10 SOS were calculated, and the values were 0.0217 (RMSE) and 0.9837 (SSIM) for the DL + 10 SOS, and 0.0221 (RMSE) and 0.9863 (SSIM) for the DL-10 SOS, while an RMSE of 0.0025 for both Theory + 10 SOS and Theory−10 SOS. The results illustrate that the values of SOS do have some effect on the performance of the DL model. The SOS was mostly used as guidance for the structure information when predicting attenuation images. The SOS image was combined with the reflection mode data to produce the attenuation images. The reflection mode data were also refraction-corrected using the SOS images. If the SOS values were changed, then the reflection mode data would also be adjusted due to refraction correction. This may explain some minor differences in the attenuation images produced when the SOS is changed by small amounts compared with the negligible changes suggested by Kramers–Kronig. Furthermore, the attenuation at the boundary changes more compared with homogeneous regions. When the SOS changes, it will affect the reflection coefficient at the boundaries, which will make the energy distribution different compared with the homogeneous regions.

### B. Validation of the Model on the In Vivo Dataset

Validation of the DL models on in vivo datasets in medical imaging is difficult. For the acoustic attenuation of the breast, the ground-truth values were not available. To perform validation of the model on in vivo dataset, we used four different approaches.

#### 1) Attenuation Values Aligned With the Literature:

The mean and variance of the attenuation values of three human breasts in this study were calculated. In each case, the ROIs were chosen to calculate the attenuation values of the fat, structural tissues (STs), and IDC. The results can be found in Table VIII. The fat region of all three cases had a mean attenuation of around 0.2 dB/cm/MHz, and the attenuation values within the ST region had a larger variance, which aligns well with the values found in the literature [40]. Furthermore, the attenuation for the IDC region was 0.88 ± 0.27 dB/cm/MHz, which was similar to the values found previously, which was 0.86 dB/cm/MHz [40] and 1.32 dB/cm/MHz [3]. The cyst region, which is characterized as mostly liquid, had a low attenuation [43]. In this study, the cyst region had a lower attenuation compared with the surrounding tissues, with a value of 0.17 ± 0.30 dB/cm/MHz.

#### 2) Segmentation Masks Between SOS-Based and DL-Based:

The correctness of the DL-based method was validated by comparing the similarities between the DL-based segmentation map and the SOS-based segmentation map. The successful capture of the cyst or cancer region and the high correlation coefficient and IoU between two segmentation maps illustrate that the DL method was valid in generating attenuation images. Moreover, the correlation coefficients were not 1, which illustrates that there were differences between two segmentation maps. The differences between two segmentation maps demonstrated that the DL-based attenuation map was able to provide additional information which was different from the SOS image. For example, from the results of the cyst case, there were more small structures shown in the DL-based segmentation map, which might provide more structure information compared with the SOS-based segmentation.

#### 3) Downsampled Version of the DL Output:

**Fig. 16.**
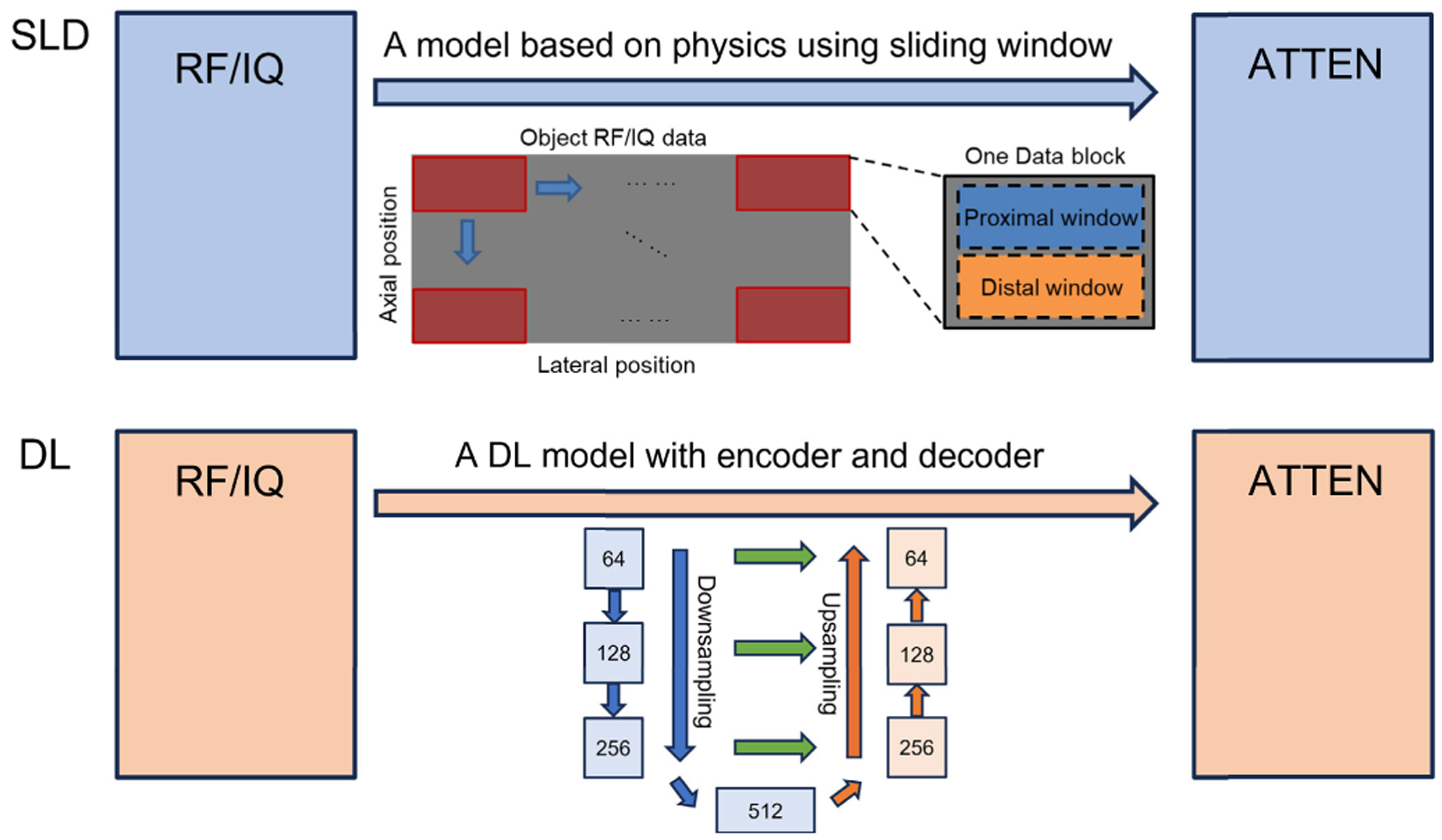
Pipeline of generating attenuation images from both the SLD and DL methods.

**Fig. 17.**
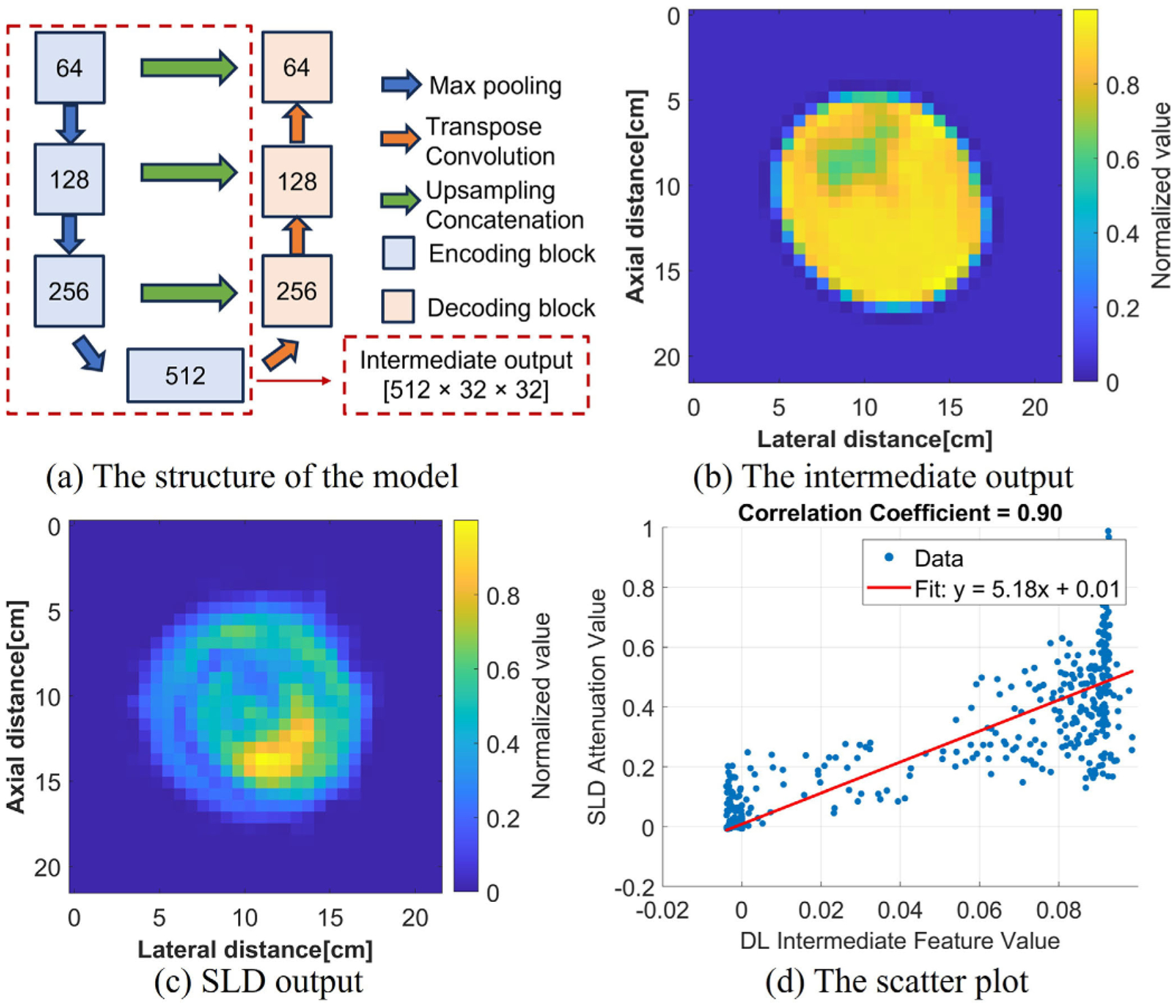
(a) Structure of the DL model, the intermediate output was extracted after the encoding blocks. (b) Intermediate output from the DL model. (c) Downsampled and normalized SLD output. (d) Scatter plot of the DL intermediate feature value and the SLD attenuation value, with a Pearson correlation coefficient of 0.9.

Two downsampled versions of the DL output were compared with the SLD output. As shown in Fig. 16, the SLD used a physics-based model using a sliding window, and this was similar to the encoding part of the DL model, which also produced a downsampled version of the input with extracted features. As a result, the intermediate output from the DL model was compared with the SLD output. The intermediate output was extracted before the decoding blocks, which was in 512 [channels] × 32 [width] × 32 [height] dimension. To get a single-channel output image, we took the mean value of all the channels. Noting that the intermediate output image did not represent the actual attenuation values, a normalization was done on the intermediate output image. To do a fair comparison, the SLD output was also downsampled and normalized to 32 × 32. The cyst case was evaluated and is shown in Fig. 17. From Fig. 17, the intermediate output from the DL looked similar to the SLD output, showing the feature of the cyst region, with other regions smoothed out, i.e., the fine structures which could be reconstructed in the final output disappeared with the intermediate output. A high correlation coefficient with 0.9 and a linear fitting with a 0.01 bias suggested that the intermediate output from the DL was highly correlated with the SLD output. The similar pattern and high correlation between the SLD and the intermediate output offered additional validation of the DL model.

**Fig. 18.**
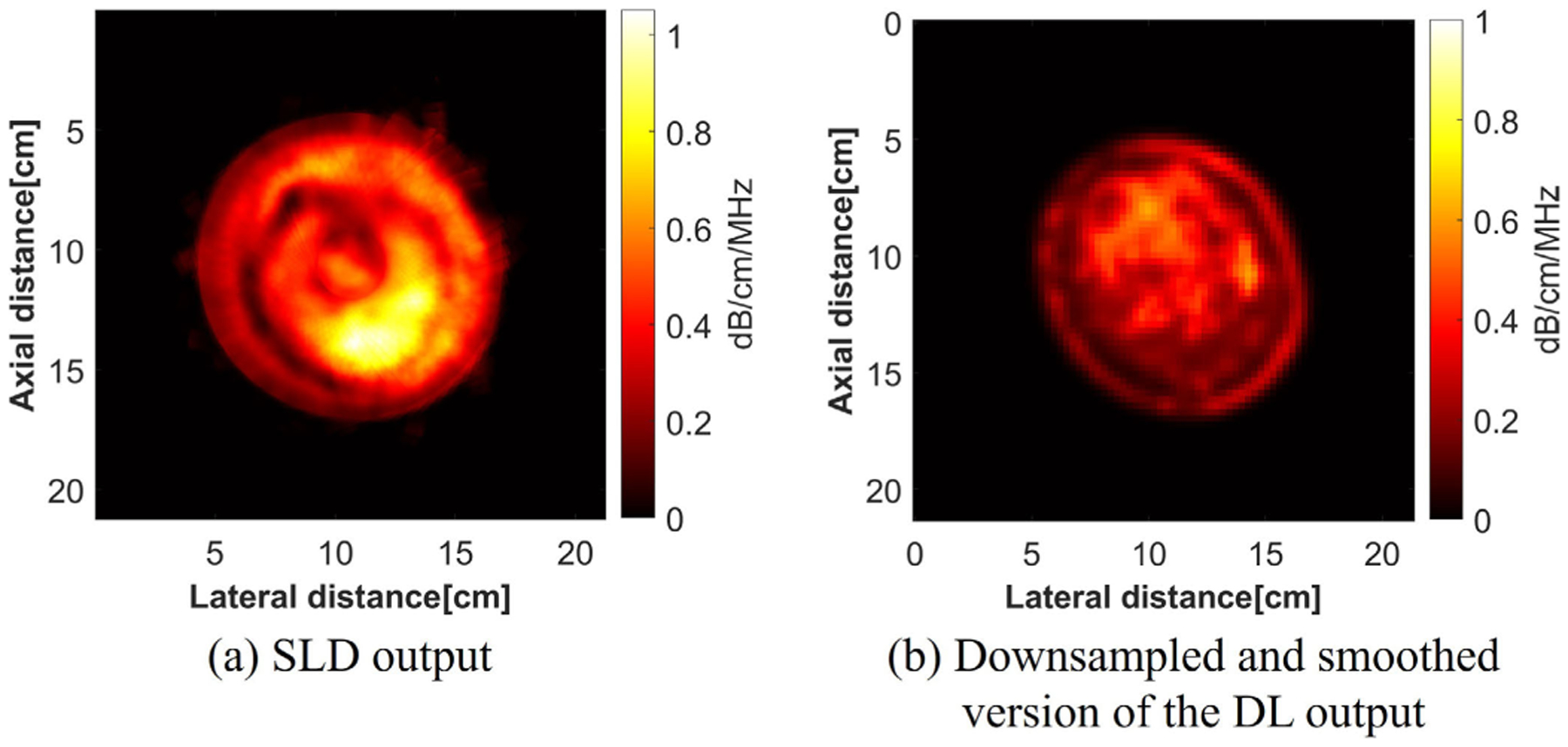
Attenuation images of the breast with a cyst. (a) SLD output. (b) DL output with downsampling and smoothing.

The final output of the DL model was not just a simple upsampled version of SLD. To make the comparison to SLD, the DL-based image was downsampled from the final output of the DL model with a Gaussian smoothing filter applied afterward. The result of the cyst case is shown in Fig. 18. It can be observed that the fine structures of the DL method were gone when doing downsampling and smoothing, and there were many similarities between the SLD and DL methods, which suggests a good match between the SLD and DL methods.

#### 4) Relationship Between SOS and Attenuation: Not a Scaled Version:

**Fig. 19.**
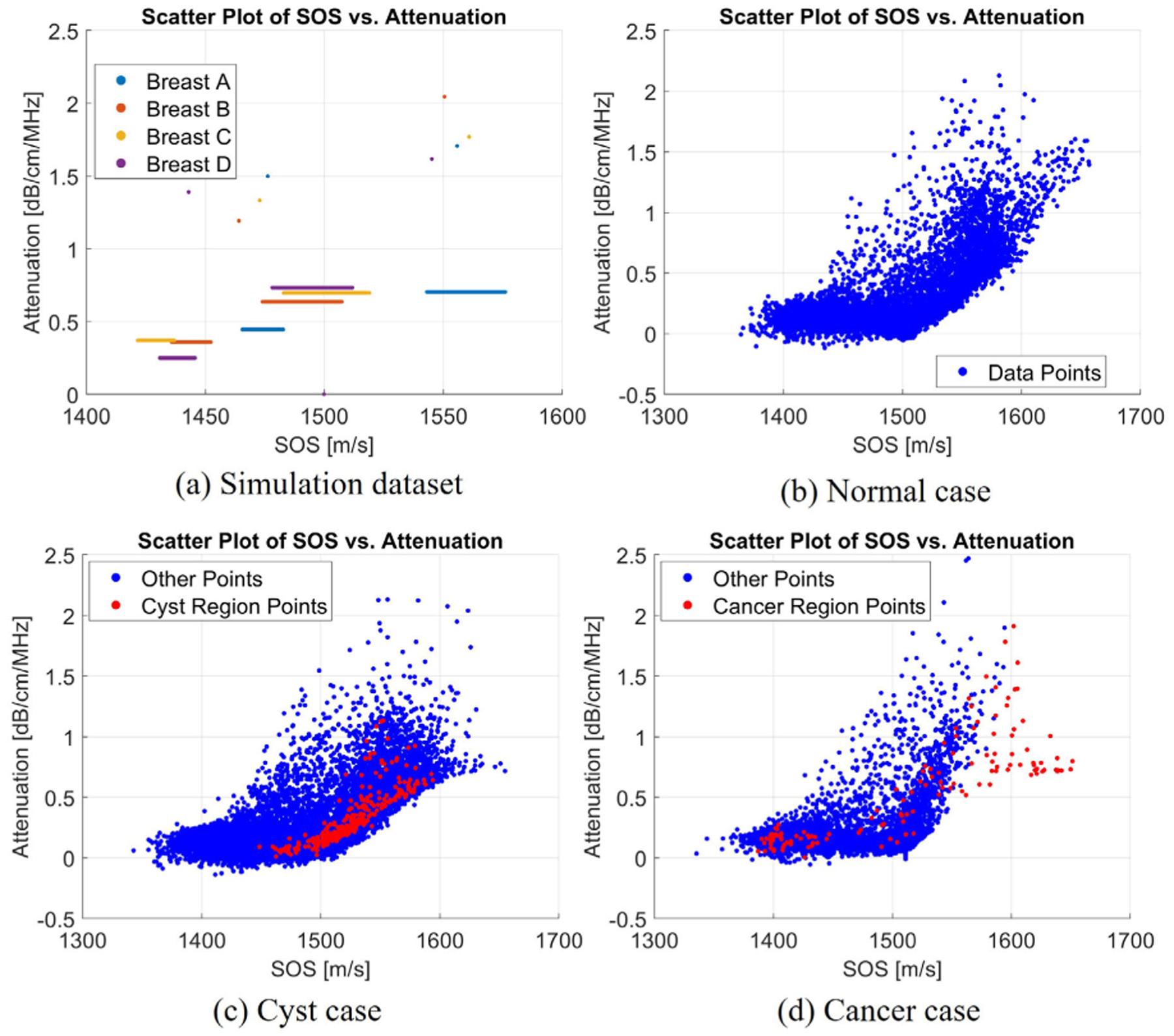
Scatter plot of the SOS and attenuation. (a) Four simulation breast datasets. (b) Normal breast. (c) Breast with a cyst. (d) Breast with cancer.

Finally, it is also worth recognizing the relationship between SOS and attenuation. The Kramers–Kronig relationships apply to phase velocity and frequency-dependent attenuation, and because group velocity (SOS) is derived from phase velocity, the SOS and attenuation are highly correlated. However, because the SOS was used as the input to train the model, it is necessary to ensure that the DL-based attenuation is not merely a scaled version of SOS. As a result, the relationship between predicted attenuation and SOS was studied. The SOS and attenuation points of three in vivo breasts and the simulation breast dataset are plotted in Fig. 19. In addition, the Spearman correlation coefficients (SCCs) of the three in vivo cases were calculated, which were −0.0606, −0.1378, and −0.3519 for the normal, cystic, and cancer breasts, respectively. For the cyst and cancer case, the cyst and cancer region’s SCC were also calculated, which were 0.9375 and 0.8381. From both the scatter plot and the correlation coefficients, the relationship between SOS and attenuation was not merely a scaled version, but had a weak linear or nonlinear relationship. The different distributions of the data between the simulation dataset and the in vivo dataset suggest that the model had a good generalization ability, not merely limited to the simulation dataset. Furthermore, the different relationship between SOS and attenuation among different types of breast can potentially be another biomarker to distinguish between normal and cancer breasts, and the high SCC in cyst and cancer region suggests a strong positive relationship between sound speed and attenuation in the abnormal regions.

### C. Ablation Study on Network Architecture

To test the effectiveness of our proposed network architecture, an ablation study on the UNET model was conducted. The model used in this study had a depth of 3 with the skip connections, which was the baseline model. For the ablation study, three other models with different depths and no skip connections were compared on the Envelope + SOS simulation dataset, which are listed in Table IX. The results indicate that when all the other settings (learning rate, batch size, etc.) are the same, as the network gets deeper (from depth 2 to depth 3), the performance becomes better. However, because of the limited size of the dataset, a too deep network does not necessarily improve the performance (from depth 3 to depth 4), but increases the number of model parameters. Furthermore, without skip connections, the performance of the model decreased.

### D. Limitations and Future Work

There are some limitations with this study. For the phantom and in vivo breast results, the background area in SOS, reflection, and predicted attenuation with the DL will have some ring artifacts and noise. However, considering that the background area is water, it is easy to do segmentation to get the breast, and then apply a mask to remove the noise in the background area. All the results with the in vivo breast had a mask applied to remove the artifacts in the background area. In addition, in terms of the test data, there was a mismatch between simulation and the physical measurements, because QTI adopts a technology called volography, which enables accurate 3-D reconstruction with ray-tracing. However, in simulation, only 2-D simulations were conducted to save time generating data. As a result, there was a mismatch between the simulation settings and test settings and only 2-D slices were analyzed in this study. Moreover, there are some other aspects that the simulation did not take into account, such as the convexity of the probe, the use of dynamic beamforming in the true data acquisition, and the correct waveform to be used. However, the superior performance of the neural network confirmed the good generalization ability of the model. Finally, because the ground-truth acoustic attenuation in the breast tissues is unknown, the predicted attenuation with the DL cannot be guaranteed to be exactly correct. However, four methods have been used to perform the validation of the DL method providing confidence that the DL-based attenuation images provide realistic data.

For future work, a 3-D volume dataset can be created and produced. Because of the limit of GPU storage and time, the training dataset was generated with 2-D slices, and the input and output of neural networks were also 2-D slices. The optimal case should be a 3-D volume image translation, and it requires a higher limit GPU storage hardware and more training time. Furthermore, the model used in this study was a simple CNN UNET architecture, and more advanced models such as the diffusion model [44] have been used widely in the normal image translation and generation field nowadays. A comparison and validation of different advanced models can be conducted in the future. In addition, more validation of the model needs to be performed with more in vivo breast data.

## V. Conclusion

In this study, a multichannel DL-based method was used to generate ultrasound attenuation images. The generated attenuation images had higher spatial resolution with lower variance than using a QUS method, i.e., SLD, and the FWI method (used by the QTI BACT scanner). The high spatial resolution attenuation images can be used to do segmentation of the human breast and provide more information for diagnosis, and the relationship between SOS and attenuation can be another biomarker to distinguish between normal and cancer breasts.

## Figures and Tables

**Figure:**
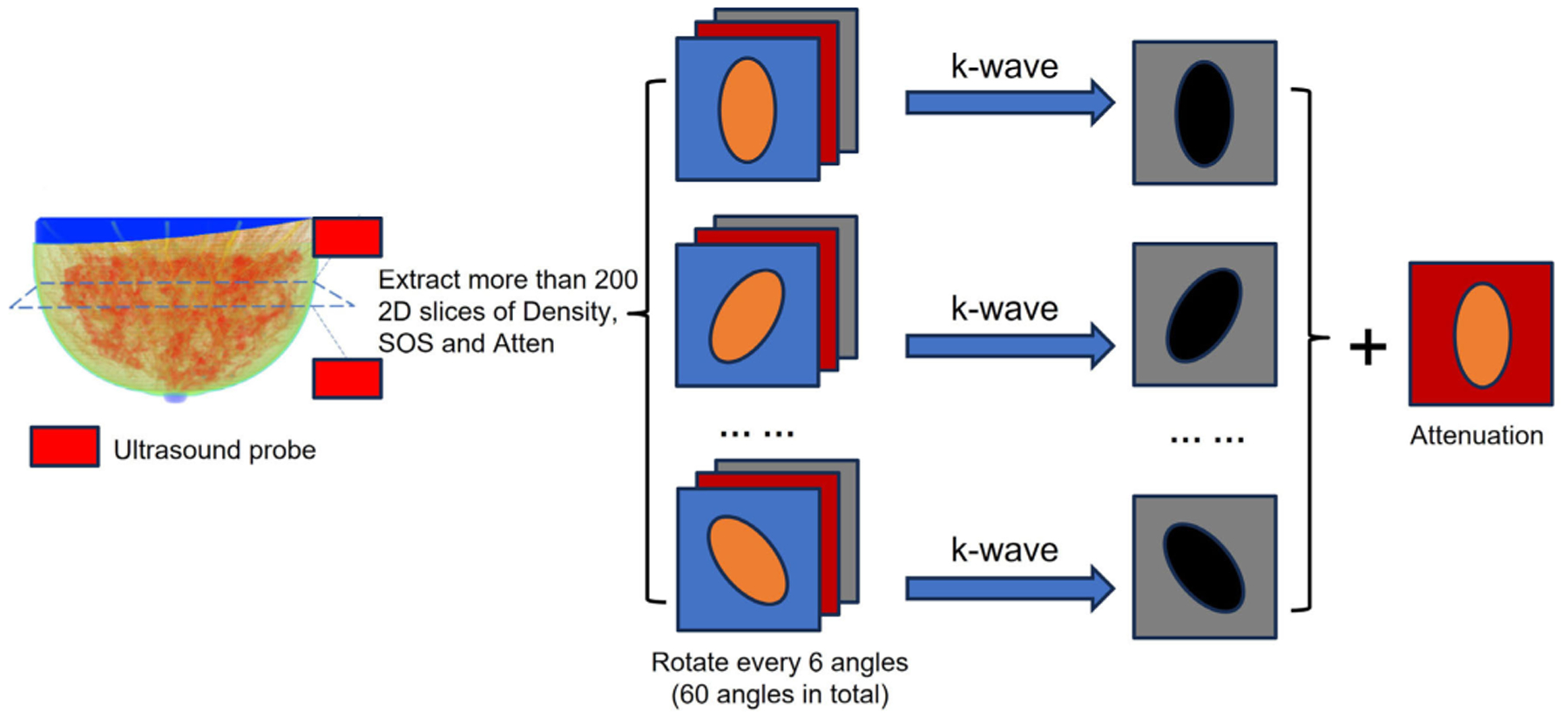
Fig. 3. Schematic of the process to generate RF signal using the 3-D digital breast phantom.

**TABLE I:** K-Wave Simulation Parameters

Transducer	Parameter settings
Center frequency	3.6 MHz
Pitch	0.4 mm
Element number	256
Transmit apodization	Hamming
Receive apodization	Rectangle
Grid size	0.1 × 0.1 mm

**TABLE II:** K-Wave Simulation Dataset

Dataset	Numbers of RF [Slice × Angle]
A	317 × 60 = 19020
B	253 × 60 = 15180
C	291 × 60 = 17460
D	235 × 60 = 14100

**TABLE III:** Dataset Composition for Neural Networks

	Breast dataset	Number of RF
Training split (A + B + D)	Slice 200–300^th^	Train: 12600 (70×60×3) Val: 3600 (20×60×3) Internal Test: 1800 (10×60×3)
External Test	C (Slice 200–300^th^)	C: 100 × 60 = 6000
	Phantom and three *in-vivo* breasts from QTI	

**TABLE IV:** Results of Simulation Dataset A + B + D

	RF	Envelope	RF + SOS	Envelope + SOS	SOS
SSIM	0.65 ± 0.03	0.60 ± 0.06	0.97 ± 0.009	**0.98 ± 0.003**	0.66 ± 0.17
RMSE	0.25 ± 0.03	0.27 ± 0.03	0.08 ± 0.004	**0.07 ± 0.007**	0.28 ± 0.09

**TABLE V:** Results of Simulation Dataset C

	RF	Envelope	RF + SOS	Envelope + SOS	SOS
SSIM	0.51 ± 0.02	0.45 ± 0.03	0.87 ± 0.01	**0.95 ± 0.002**	0.63 ± 0.03
RMSE	0.29 ± 0.006	0.30 ± 0.009	0.17 ± 0.006	**0.11** ± **0.001**	0.31 ± 0.01

**TABLE VI:** Results of the Homogeneous Phantom Experiments

	FWI	DL	SLD	Ground-truth
Value	0.87 ± 0.10	**0.69 ± 0.05**	-	0.76

**TABLE VII:** Attenuation Results of Nine Breasts With Cancer

	Pathology	Mean Att	Std Att
(a)	IDC	0.98	0.21
(b)	ILC	0.73	0.33
(c)	IDC	0.47	0.12
(d)	IDC	0.48	0.17
(e)	Mixed IDC and MetCa	0.54	0.16
(f)	IDC	0.68	0.46
(g)	Mixed IDC, ILC and MucCa	0.43	0.33
(h)	Mixed IDC and ILC	0.50	0.20
(i)	IDC	0.51	0.13
Average		0.59	0.25

**TABLE VIII:** Attenuation Values of the In Vivo Breast Samples

Breast type	Fat	ST	IDC	Cyst
Normal	0.18 ± 0.09	0.37 ± 0.30	-	-
Cyst	0.20 ± 0.19	0.56 ± 0.09	-	0.17 ± 0.30
Cancer	0.19 ± 0.05	0.36 ± 0.33	0.88 ± 0.27	-

**TABLE IX:** Results of the Ablation Study on Different Network Architectures on the Simulation Dataset A + B + D

Model	Depth	Skip	SSEM	RMSE	Params (M)
shallow	2	**✓**	0.96	0.09	1.90
baseline	3	**✓**	**0.98**	**0.07**	7.73
deep	4	**✓**	0.96	0.09	31.07
baseline-noskip	3	**✗**	0.58	0.27	6.96

## Highlights

- A DL-driven high spatial resolution attenuation imaging was proposed with the help of the QT Imaging Breast Acoustic CT scanner.
- By including the high-resolution sound speed images from the BACT scanner, the DL-generated attenuation images had improved spatial resolution compared with traditional methods, i.e., QUS and FWI.
- The high spatial resolution DL-generated attenuation images can be used as another biomarker to assist in breast cancer diagnosis.

## Footnotes

1 Trademarked.

## Appendix

Deriving from the Kramers–Kronig relationship under the exponent term equal to 1, we have

(A1)
$$\frac{1}{c\left(\omega \right)}-\frac{1}{c\left(\omega_{0}\right)}=-\frac{2}{\pi }\alpha_{0}\text{ln}\left|\frac{\omega }{\omega_{0}}\right|$$

where c(ω) denotes the phase velocity, and $\alpha_{0}$ denotes the attenuation. Taking the derivative with respect to ω on both sides, we can get

(A2)
$$\frac{d}{d\omega }\left(\frac{1}{c_{p}\left(\omega \right)}\right)=-\frac{2}{\pi }\frac{\alpha_{0}}{\omega }.$$

The SOS, which is the group velocity, is defined as

(A3)
$${\left.c_{g}\left(\omega \right)={\left(\frac{d}{d\omega }\text{Re}\left[k\left(\omega \right)\right]\right)}^{-1}=\left(\frac{d}{d\omega }\left(\frac{\omega }{c_{p}\left(\omega \right)}\right)\right)\right)}^{-1}.$$

In addition, by simplifying the term $(d/d\omega )\left(\left(\omega /c_{p}(\omega )\right)\right)$, we can get

(A4)
$$\frac{d}{d\omega }\left(\frac{\omega }{c_{p}\left(\omega \right)}\right)=\frac{1}{c_{p}\left(\omega \right)}+\omega \frac{d}{d\omega }\left(\frac{1}{c_{p}\left(\omega \right)}\right).$$

Substituting with (A2) in (A3), we get

(A5)
$$\frac{1}{c_{g}\left(\omega \right)}=\frac{1}{c_{p}\left(\omega \right)}-\frac{2}{\pi }\alpha_{0}.$$

Moreover, by simplifying (A3), we can get

(A6)
$$c_{g}\left(\omega \right)=\frac{c_{p}^{2}\left(\omega \right)}{c_{p}\left(\omega \right)-\omega \frac{dc_{p}\left(\omega \right)}{d\omega }}.$$

In soft tissues, the dispersion is weak, i.e., $\left(dc_{p}/d\omega \right)$ is small. As a result, we can get

(A7)
$$c_{g}\left(\omega \right)=c_{p}\left(\omega \right)\left(1+\omega \frac{dc_{p}}{c_{p}\left(\omega \right)d\omega }\right)=c_{p}\left(\omega \right)+\omega \frac{dc_{p}}{d\omega }.$$

Now when both the group velocity and phase velocity have a small perturbation, we have

(A8)
$$\Delta c_{g}=\Delta c_{p}+\Delta \left(\frac{dc_{p}}{d\omega }\right).$$

Because $\Delta \left(\left(dc_{p}/d\omega \right)\right)$ is second-order small, we can assume $\Delta c_{g}\approx \Delta c_{p}=\Delta$. Then, using the Taylor expansion for both $c_{p}$ and $c_{g}$ supposing a small perturbation Δ

(A9)
$$\frac{1}{c_{p}+\Delta }\approx \frac{1}{c_{p}}-\frac{\Delta }{c_{p}^{2}}$$

(A10)
$$\frac{1}{c_{g}+\Delta }\approx \frac{1}{c_{g}}-\frac{\Delta }{c_{g}^{2}}.$$

Subtracting (A9) with (A10), we can get

(A11)
$$\alpha_{0}^{\prime }=\alpha_{0}+\frac{\pi }{2}\Delta \left(\frac{1}{c_{g}^{2}}-\frac{1}{c_{p}^{2}}\right).$$

Substituting $c_{p}$ using (A5), and simplifying the equation, we can get the new attenuation $\alpha_{0}^{\prime }$ in unit Np/m/Hz

(A12)
$$\alpha_{0}^{\prime }=\alpha_{0}\left(1-\frac{2\Delta }{c_{g}}\right)-\frac{2\Delta }{\pi }\alpha_{0}^{2}.$$
